# Teneurin-2 and related proteins in reactive astrocytes after *status epilepticus* induction in adult rats

**DOI:** 10.3389/fnins.2025.1670634

**Published:** 2026-03-18

**Authors:** Gestter Willian Lattari Tessarin, David William Hogg, Alaide Gonçalves, Daniel Vieira Casatti, Vitória Morelli Savenhago, Bárbara Stéfani Silva da Costa, Viviam Barbuglio Del Priore, David Alan Lovejoy, José Anchieta de Castro Horta-Junior, Cláudio Aparecido Casatti

**Affiliations:** 1 Department of Basic Sciences, School of Dentistry of Araçatuba, São Paulo State University (UNESP), Araçatuba, São Paulo, Brazil; 2 Division of Anatomy, Department of Functional and Structural Biology, Institute of Biosciences of Botucatu, São Paulo State University (UNESP), Botucatu, São Paulo, Brazil; 3 School of Dentistry, University Center in the North of São Paulo (UNORTE), São José do Rio Preto, São Paulo, Brazil; 4 Department of Cell and Systems Biology, University of Toronto, Toronto, ON, Canada

**Keywords:** adhesion G protein-coupled receptor, adult rat, epilepsy, pilocarpine, reactive astrocytes, *status epilepticus*, TCAP, teneurin

## Abstract

**Introduction:**

Teneurins are a protein family composed of four paralogs in vertebrates (Ten-1-4), expressed mainly in the central nervous system (CNS), which primarily participate in neuronal circuit establishment through homophilic or heterophilic interactions. These proteins possess a bioactive peptide, teneurin C-terminal associated peptide (TCAP1-4), located at the distal extracellular tip. The adhesion G protein-coupled receptors L (ADGRL1-3) are the main receptors for teneurins. The purpose of this study was to evaluate the Ten-2/TCAP-2/ADGRL1 system in the cerebral cortex and hippocampus in a rat model of epilepsy.

**Methods:**

Adult male rats received LiCl-pilocarpine and were euthanized 2, 5, 14, 35 and 65 days after *status epilepticus* (SE) induction. Ten-2, TCAP-2 and ADGRL1 immunohistochemistry and quantitative gene expression were performed in the primary somatosensory area and hippocampus. Fluoro-Jade C staining was used to verify colocalization between neuronal degeneration and immunoreactive cells.

**Results:**

Increases in Ten-2- (Ten-2-LI) and ADGRL1-like immunoreactive (ADGRL1-LI) reactive astrocyte profiles were mainly found in the primary somatosensory (5 days after SE, *p* < 0.0001) and CA3 (2, 5 and 14 days, *p* < 0.0001) areas, when compared with other groups. Ten-2, TCAP-2 and ADGRL1 gene expressions showed significant up-regulation in the cerebral cortex (5 days, *p* < 0.001) and CA3 (2 and 5 days, *p* < 0.0001). Ten-2-LI and ADGRL1-LI were colocated in the same reactive astrocyte profiles, positioned in brain regions with neuronal degeneration.

**Conclusion:**

This study demonstrated that SE induced an up-regulation of Ten-2, TCAP-2 and ADGRL1 in reactive astrocytes of adult rats, indicating that the astrocytic teneurin-ADGRL system is modulated after *status epilepticus* induction.

## 1 Introduction

Teneurins are single-pass transmembrane glycoproteins present as four paralogs in vertebrates (Ten-1-4), mostly expressed in neurons during neurogenesis. This group of proteins are functionally involved with neuronal migration, axonal orientation, cell signaling, development and structural maintenance of synapses, myelination, among other functions (Baumgartner and Chiquet-Ehrismann, 1993; Minet et al., 1999; Rubin et al., 1999; Tucker and Chiquet-Ehrismann, 2006; Suzuki et al., 2012; Mosca, 2015; Li et al., 2018; Tucker, 2018; Zhang et al., 2022; Zhang et al., 2025). Structurally, teneurins present the carboxyl (C-terminal) and amino (N-terminal) domains with extra and intracellular orientation, classified as type II transmembrane proteins, exhibiting extracellular sites for hemophilic and/or heterophilic protein interactions (Tucker and Chiquet-Ehrismann, 2006; Rubin et al., 1999; Feng et al., 2002; Leamey et al., 2007; Mosca, 2015; Tucker, 2018). The C-terminal domain of all teneurins has a cleavage site that may produce a peptide of 40 to 41 amino acids, denominated as “teneurin C-terminal associated peptide” (TCAP-1-4) (Lovejoy et al., 2006). TCAP-1 functional activities have been explored in several *in vivo* and *in vitro* studies, indicating significant effects in stress modulation induced by corticotrophin-related factor (CRF), neuronal cytoskeletal rearrangements permitting axonal growth and dendritic arborization, neuroprotection, oxidative stress modulation, stimulation of uptake of glucose by neurons as well as reduced neuronal intracellular calcium (Wang et al., 2005; Lovejoy et al., 2006; Al Chawaf et al., 2007; Trubiani et al., 2007; Tan et al., 2009; Tan et al., 2011; Woelfle et al., 2015; Hogg et al., 2018; Hogg et al., 2019; Hogg et al., 2022).

The latrophilin-1-associated synaptic surface organizer (Lasso), a Ten-2 splice variant, exhibits heterophilic interactions with the adhesion G protein-coupled receptor L (ADGRL) family (Silva et al., 2011). The ADGRL family consists of three paralogs (ADGRL1, ADGRL2 and ADGRL3) also known as latrophilins (Silva et al., 2011; Sita et al., 2019). ADGRL is a calcium-independent receptor for alpha-latrotoxin (Silva et al., 2011; Vysokov et al., 2016). Functionally, the interaction between teneurins and ADGRL promotes synapse stabilization, alterations in the influx of presynaptic intracellular calcium and changes in cyclic AMP (cAMP) (Silva and Ushkaryov, 2010; Silva et al., 2011; Boucard et al., 2014; Vysokov et al., 2016; Li et al., 2018; Vysokov et al., 2018).

Epilepsy is a chronic brain disorder where an electrophysiological dysfunction occurs, providing the appearance of induced or spontaneous epileptic seizures (Stafstrom and Carmant, 2015; Devinsky et al., 2018; Patel et al., 2019). In this pathology, there is an imbalance between excitatory and inhibitory signaling in certain areas of the brain with a neurotransmitter disorder, promoting changes in cellular activities that can spread to different areas of the central nervous system (CNS), resulting in epileptic or seizure conditions (Goldberg and Coulter, 2013; Akyuz et al., 2021). Astrocytes take part in nervous tissue homeostasis, regulation of neuronal excitability and plasticity, metabolization of neurotransmitters (e.g., glycine, glutamate and GABA), as well as secretion of neuroactive molecules, such as gliotransmitters and neurotrophic factors (Araque et al., 1999; Sofroniew and Vinters, 2010; Araque et al., 2014; Magaki et al., 2018). In addition, the connection of the astrocytic endfeet with blood vessels is essential for the formation and maintenance of the blood–brain barrier (BBB), contributing to the selectivity of the influx of certain molecules toward the CNS (Janzer and Raff, 1987; Abbott et al., 2006; Sofroniew and Vinters, 2010; Manu et al., 2023). Astrocytes also play an essential role in CNS dysfunction, such as traumatic brain injury, neuroinflammation, viral infection, neurodegenerative disorders, and epilepsy (Wetherington et al., 2008; Losi et al., 2012; Sofroniew, 2014; Magaki et al., 2018; Neal and Richardson, 2018; Vargas-Sánchez et al., 2018; Sofroniew, 2020; Todd and Hardingham, 2020; Verkhratsky et al., 2023). After seizures and/or in epilepsy disorders, astrocytes undergo changes in their morphology, physiology, and biochemistry to support proper function and to modulate neuronal activity (Wetherington et al., 2008; Losi et al., 2012; Vargas-Sánchez et al., 2018; Todd and Hardingham, 2020; Verhoog et al., 2020; Heuser and Enger, 2021; Twible et al., 2021; Purnell et al., 2023). For example, after excessive neuronal excitation, glutamate, sodium and potassium uptake from the intercellular space by astrocytes is important to prevent or minimize neuronal excitotoxicity and associated oxidative stress (Vargas-Sánchez et al., 2018; Todd and Hardingham, 2020; Verhoog et al., 2020). Particularly, the calcium signaling is significantly changed in these disorders (Scorza et al., 2009; Heuser and Enger, 2021; Koizumi et al., 2021).

The first study to link teneurins with neuroplasticity during central nervous system (CNS) injury was conducted by Otaki and Firestein (1999), who reported Ten-2 re-expression in regenerating external tufted cells following chemical damage to the nasal mucosa. Subsequent studies has shown altered Ten-2 gene expression in ischemic stroke, schizophrenia, and Alexander disease, the latter being characterized by astrocytic alterations resulting from mutations in the GFAP gene (Zamanian et al., 2012; Kondo et al., 2016; Li et al., 2021). A significant increase in Ten-2 levels in reactive astrocytes has also been observed after mechanical injury to the cerebral cortex (Tessarin et al., 2019). More recently, mutations in ADGRL1 have been associated with brain functional disorders, including epilepsy, in both animal models and human patients (Vitobello et al., 2022; Lei et al., 2025). Based on these data, the present study assessed whether Ten-2, TCAP-2 and ADGRL1 are present in reactive astrocytes in the cerebral cortex and hippocampus, in a condition where neuronal hyperactivity and calcium oscillations are harmful through the lithium chloride-pilocarpine (LiCl-pilocarpine) rat model to induce *status epilepticus* (SE) and epilepsy.

## 2 Materials and methods

### 2.1 Animals

Adult male Wistar rats (*Rattus novergicus*, 280–300 body weight; approximately 60 days old) from the central animal facility at the School of Dentistry of Araçatuba (UNESP, Araçatuba, SP, Brazil) were used in this study. Initially, the animals were maintained in cages (4 or 5 animals per cage) in the experimental area of the Morphology division of the Department of Basic Sciences for 15 days under controlled conditions of 12/12 h light–dark cycle (light cycle from 7:00 a.m. to 7:00 p.m.), a room temperature of 22° C (± 2° C), relative humidity of 50%, with access to water and chow *ad libitum* for environmental adaptation. All the experimental procedures were conducted according to the guidelines for care and use of mammals in neuroscience and behavioral research (National Research Council (US) Committee on Guidelines for the Use of Animals in Neuroscience and Behavioral Research, 2003) and approved by the local animal care and use committee (CEUA; FOA process number 0847/2018, School of Dentistry of Araçatuba, UNESP, Araçatuba, SP, Brazil). Animal health was supervised by the veterinarian or their appointed animal care technicians of our university animal health care unit. In addition, our own trained researchers, graduate and undergraduate students (GWLT, AG, BSSC, DVC, and VSM), as well as technicians reviewed animal behavior and general health every day. The animals were humanely euthanized via intramuscular injection of one-two doses of ketamine (70 mg/kg, Francotar®, Virbac, SP, Brazil) and xylazine (10 mg/kg, Rompum®, Bayer, RS, Brazil) by the veterinarian staff, if the animals displayed more than 20% weight loss and significant deterioration in general health. In this study, 42 animals were lost during the first 48 h after SE induction due to cardiorespiratory arrest and a few animals (*n* = 7) were humanely euthanized. Forty-nine animals (*n* = 49) were used in all seven experimental groups (*n* = 7 animals for each group) permitted all counting for statistical analysis and did not affect the findings of the present study.

### 2.2 Induction of *status epilepticus* (SE) using LiCl-pilocarpine model

First, the animals from all groups (except naïve group - NAG) were submitted to intraperitoneal (i.p.) injection of lithium chloride (LiCl; 1,519, Dinâmica Química Contemporânea, SP, Brazil; 127 mg/kg diluted in saline solution) 18–20 h before SE induction to potentialize and reduce the pilocarpine dose used in each animal. Next, 30 min before pilocarpine administration, methyl scopolamine bromide (i.p., 1 mg/kg, S8502, Sigma-Aldrich, MO, USA) was administered to reduce the cholinergic pilocarpine effects. Subsequently, *status epilepticus* (SE) was induced with pilocarpine hydrochloride (i.p., 40 mg/kg diluted in saline solution, P6503, Sigma-Aldrich, MO, USA) (Wu et al., 2018; Osuntokun et al., 2021). From this moment on, the animals were supervised, and visually monitored to analyze the behavioral signs of SE. The SE was maintained for 2–3 h and ceased with administration of diazepam (i.p., 10 mg/kg; Teuto, GO, Brazil) once and/or repeatedly if convulsive changes persisted. After these steps, the animals were maintained in cages (1 animal per cage), rehydrated with intradermal glycosylated saline solution (between day 0 and day 3 after SE) and creamy chow mixture was administered to facilitate ingestion in some animals with reduced feeding ability. Next, the animals were progressively selected into five epilepsy groups (seven animals per group, *n* = 7): 2, 5, 14, 35 and 65 days (epilepsy groups: EG2, EG5, EG14, EG35 and EG65). Only animals classified as grade 5 on Racine scale were included in this study (Racine, 1972). Animals making up EG35 and EG65 groups were continuously video monitored and were euthanized for 5 days after exhibiting spontaneous seizures classified as grade 5 on Racine scale (around 35 or 65 days). Pharmacological control group animals (PCG, *n* = 7) received administration of lithium chloride, methyl scopolamine bromide, saline solution without pilocarpine and one dose of diazepam. The naïve group (NAG, *n* = 7) was not submitted to any procedure. Animals from PCG were euthanized 5 days after solution administrations, as the main changes in Ten-2/TCAP-2/ADGRL1 were noticed in animals from epilepsy group euthanized 5 days after SE induction. Animals from NAG were maintained in the same experimental room for the same 5-day period without handling, before the euthanized.

### 2.3 Histological brain tissue analysis of hippocampus and cerebral cortex

To analyze the hippocampus (CA3 region) and cerebral cortex (primary somatosensory area), four animals (*n* = 4) for each group were anesthetized by intramuscular injection of ketamine (70 mg/ kg, Francotar®, Virbac, SP, Brazil) and xylazine (10 mg/kg, Rompum®, Bayer, RS, Brazil) and submitted to transcardiac perfusion with 100 mL of heparinized (0.1%, Hemofol® 5,000 U. I., SKUP6242, Cristália, SP, Brazil) saline solution and 800 mL of 4% formaldehyde (76,240, Sigma-Aldrich, MO, USA) diluted in phosphate-buffered saline (PBS) 0.1 M pH 7.4. After this step, the rat brains were removed, dissected and post-fixed in the same fixative solution (4% formaldehyde) for 4 h at 4 °C. Then, the brains were subjected to cryoprotection procedures using 30% sucrose solution diluted in PBS 0.1 M pH 7.4 for 48 h. All cryoprotected brains were sectioned at 40 μm thickness in coronal plane using a conventional microtome (SM 2000R, Leica, HE, Germany) adapted for freezing. The sections were then stored in two 12-well culture plates with an anti-freezing solution (30% ethylene glycol - 00E1020. 06. BC, Synth, SP, Brazil; 20% glycerol - 5092-08, Synth, SP, Brazil) diluted in PBS 0.1 M pH 7.4. These histological sections were submitted to Nissl staining, immunohistochemistry (indirect immunofluorescence and immunoperoxidase) and Fluoro-Jade C staining.

### 2.4 Nissl stain

One histological series of sections (40 μm thickness) was mounted on gelatinized (0.4% gelatin, G9391, Sigma-Aldrich, USA; 0.06% chromium and potassium sulfate, 243,361, Sigma-Aldrich, USA diluted in 0.1 M PBS pH 7.4) slides, dried at room temperature and submitted to Nissl staining to obtain detailed areas and subdivisions of the rat brain. Briefly, the sections were rinsed in a mixture containing distilled water and 10% paraformaldehyde for about 30 min. The sections were then rinsed in distilled water, dehydrated in ethanol (from 50° GL to 100° GL – 1 min each), rinsed in distilled water for 1 min and incubated in 0.25% thionin solution (88,930, Sigma-Aldrich, USA) with 1% acetic acid (A6283, Sigma-Aldrich, USA), diluted in PBS 0.1 M pH 4.5 for 1 min at room temperature. Finally, the histological sections were dehydrated in increasing series of alcohols (50, 70, 90, 95% and three times in 100%) for 5 min each, clarified in xylene (three washes of 10 min each) and covered in mounting medium (Entellan, 107,961, Merckmillipore, HE, Germany) and a glass coverslip.

### 2.5 Immunohistochemistry

#### 2.5.1 Indirect double immunofluorescence

One histological series (480 μm intersection interval) of sections (40 μm thickness) was submitted to this technique to obtain details of the morphology of astrocytes and to analyze whether this type of cells express Ten-2-LI or ADGRL1 after SE in all experimental time points adopted in this study. For this, the sections were washed (3 times for 1 min) in PBS to remove the anti-freezing solution and then blocked for non-specific bindings using a mix of PBS, 5% non-fat milk and 0.03% triton X-100 (PBS-TX, 100882547, X-100, Sigma-Aldrich, MO, USA) for 1 h. Again, another blocking for non-specific bindings was performed using a solution with 3% bovine serum albumin (A9647, Sigma-Aldrich, MO, USA) diluted in PBS-TX for 1 h, followed by 2% normal donkey serum (017–000-121, Jackson Immunoresearch, PA, USA) diluted in PBS-TX for 24 h at 4 °C. Next, the sections were incubated in a solution of primary polyclonal antibody anti-Ten-2 (1:100, Lot K1910, sc-165674, Santa Cruz Biotechnology, CA, USA) or ADGRL1 (1:1000, Lot ALR021AN0102, ALR-021, Alomone Labs, Jerusalem, Israel) PBS-TX and 2% normal donkey serum for 48 h at 4 °C with slow agitation. After this step, the sections were incubated in the specific biotinylated secondary antibody (1:800, lot #G0815, sc-2042, Santa Cruz Biotechnology or 1:500, lot #1515108, 711–065-152, Jackson Immunoresearch Laboratories Inc., PA, USA) for 90 min at 4° C followed by Cy3-streptavidin incubation (1:500, #016–160-084, Jackson Immunoresearch, PA, USA) for 60 min at 4 °C. Next, the sections were washed in PBS and dipped in a solution with PBS-TX, 2% normal donkey serum and primary polyclonal antibody anti-GFAP (1:250, Lot #2145934, AB5804, EMD Millipore, MA, USA or 1;500, Lot #2987471, AB5541, EMD Millipore Corporation, CA, USA), overnight at 4 °C. After that, they were again incubated in FITC-conjugated secondary antibody (1:200, Lot # I1213, sc-2090, Santa Cruz Biotechnology or 1:200, Lot 152,661, 703–095-155, Jackson ImmunoResearch Laboratories Inc., PA, USA) and PBS. Finally, the brain tissues were counterstained with DAPI (TR-100-FJ, Biosensis, SA, Australia) diluted in PBS according to the manufacturer’s instructions, mounted on gelatinized slides and coverslipped with buffered glycerol.

#### 2.5.2 Triple indirect immunofluorescence

To confirm that reactive astrocytes (GFAP) exhibit Ten-2- and ADGRL1-LI simultaneously, some histological sections (40 μm thickness) were submitted to triple indirect immunofluorescence and confocal microscopy analysis. Briefly, brain histologic sections (one section from each animal) were initially blocked for non-specific bindings using a mix of PBS, 5% non-fat milk and 0.03% PBS-TX for 1 h. Additional blocking was performed using a solution with 3% bovine serum albumin (A9647, Sigma-Aldrich, MO, USA) diluted in PBS-TX for 1 h followed by 2% normal donkey serum (017–000-121, Jackson Immunoresearch, PA, USA) diluted in PBS-TX for 24 h at 4 °C. The sections were incubated in a solution of primary polyclonal antibody anti-Ten-2 raised in sheep (1:1000, CAH00223061, AF4578, R&D Systems, MN, USA), ADGRL1 raised in rabbit (1:1000, Lot ALR021AN0102, ALR-021, Alomone Labs, Jerusalem, Israel) and GFAP raised in chicken (1:500, Lot # 2987471, AB5541, EMD Millipore Corporation, CA, USA), diluted in PBS-TX and 2% normal donkey serum for 48 h at 4 °C with slow agitation. After this step, the sections were incubated with FITC-conjugated secondary antibody donkey anti-chicken (1:800, lot # 152661, 703-095-155, Jackson Immunoresearch Laboratories Inc., PA, USA), Cy3-conjugated secondary antibody donkey anti-rabbit (1:500, lot #154879, 711-165-152, Jackson Immunoresearch Laboratories Inc., PA, USA) and biotinylated secondary antibody donkey anti-sheep (1:500, lot #110095, 713-065-147, Jackson Immunoresearch Laboratories Inc., PA, USA) for 90 min at room temperature. Finally, the sections were incubated with Alexa fluor 647-conjugated streptavidin (1:500, lot#154016, 016-600-084, Jackson Immunoresearch Laboratories, Inc., PA, USA) for 90 at room temperature and counterstained with DAPI (TR-100-FJ, Biosensis, SA, Australia). The sections were washed in PBS and coverslipped with buffered glycerol. The sections were then analyzed in confocal microscope.

#### 2.5.3 Indirect immunoperoxidase

One histological series of section (40 μm thickness) was submitted to an immunoperoxidase technique for detection of Ten-2 or ADGRL1 immunoreactivity to analyze the possible alterations in immunoreactivity density. Thus, the sections were washed in PBS as described above and treated with 0.3% hydrogen peroxide (H3410, Sigma-Aldrich, MO, USA) diluted in PBS for 30 min. Next, blockings for non-specific bindings were equally performed for the immunofluorescence technique. After that, the sections were incubated in the primary polyclonal antibody, anti-Ten-2 (1:100, Lot # K1910, sc-165674, Santa Cruz Biotechnology, CA, USA), or ADGRL1 (1:3000, Lot ALR021AN0102, ALR-021, Alomone Labs, Jerusalem, Israel), PBS-TX and 2% normal donkey serum for 48 h at 4 °C, followed by the specific biotinylated secondary antibody (1:800, lot # G0815, sc-2042, Santa Cruz Biotechnology or 1:500, lot #1515108, 711-065-152, Jackson Immunoresearch Laboratories Inc., PA, USA) for 90 min at 4 °C. Next, the sections were treated with avidin-biotin complex (1:500, PK-6100, Vector Laboratories Inc., CA, USA) diluted in PBS for 2 h. The reaction was then developed using a solution with 0.05% diaminobenzidine as a chromogen (DAB, 32741, Sigma-Aldrich, MO, USA), nickel ammonium sulfate (N48-500, Fisher Chemical, NJ, USA) and 0.3% hydrogen peroxidase under light microscope for visual immunoreaction control. Finally, the specimens were mounted on gelatinized slides, dehydrated in alcohol, diaphanized in xylenes and cover-slipped with DPX medium (06522, Sigma-Aldrich, MO, USA). The immunoreactions for all groups were developed using the same amount of time to avoid alterations in immunoreactivity density.

#### 2.5.4 Immunohistochemistry control

The immunohistochemistry controls for immunoperoxidase staining or double immunofluorescence were performed using primary or secondary antibody omissions. In addition, adsorption tests were performed using control peptide (Ten-2, Lot # E1011, sc-165674P, N-13, 100 μg/0.5 mL, Santa Cruz Biotechnology, CA, USA) at different concentrations (1:1; 1:0.1; 1:0.01; 1:0.005) (Tessarin et al., 2019). This control reaction was previously published (Torres-da-Silva et al., 2017; Tessarin et al., 2019). The immunolabeled cells in this study were described as Ten-2-, GFAP- or ADGRL1-like-immunoreactive neurons or reactive astrocytes, as it is not possible to affirm whether the immunopositivity comes from antibody interaction with epitopes present in the wild protein, splice variants or protein fraction generated after proteolysis.

#### 2.5.5 Indirect immunofluorescence combined with Fluoro-Jade C staining

To determine whether the Ten-2-LI reactive astrocytes in the cerebral cortex and hippocampus are co-located in areas exhibiting neuronal degeneration, one series of histological sections (40 μm thickness) was initially submitted to immunofluorescence technique, as described above, followed by Fluoro-Jade C staining using a commercial kit (TR-100-FJ, Biosensis®, SA, Australia). For these studies, the sections were mounted on gelatinized slides (after immunohistochemistry), dried at room temperature for 24 h and maintained in a histological oven for approximately 1 h at 55–56 °C. Next, the specimens were rinsed in 1% sodium hydroxide diluted in 80% ethanol for 5 min and incubated in 70% ethanol and distilled water for 2 min each, respectively. After that, the slices were treated with 0.006% potassium permanganate diluted in distilled water for 10 min. Again, the sections were rinsed in distilled water and incubated in 0.0001% Fluoro-Jade C solution for 10 min. Lastly, the sections were washed in distilled water for 1 min, counterstained with DAPI (from Fluoro-Jade C staining kit), dried at room temperature in a dark chamber and cover-slipped with buffered glycerol.

#### 2.5.6 Immunohistochemical analysis

The selected brain areas from histological sections (40 μm thickness) were submitted to double immunofluorescence (Ten-2-LI/GFAP or ADGRL1-LI/GFAP) or immunofluorescence associated with Fluoro-Jade C staining (Ten-2-LI/Fluoro-Jade) were qualitatively analyzed using a confocal laser scanning microscope (TCS-SP5 AOBS Tandem Scanner, LEICA, HE, Germany) coupled to an inverted optical microscope (Leica DMI 6000CS) at the Electron Microscopy Center of the Institute of Biosciences of Botucatu (IBB-UNESP, Botucatu, SP, Brazil) and the background was adjusted in real time to control the sensitivity and non-specificity.

Histological sections submitted to triple indirect immunofluorescence were qualitatively analyzed using a confocal laser scanning microscope (TCP-SP8 AOBS Tandem Scanner, LEICA, HE, Germany) with specific laser lines under 10 × and 20 × magnifications (Experimental Research Unit – UNIPEX, Faculty of Medicine of Botucatu - FMB/UNESP). The images were analyzed using LAS X software AND saved in digital files. A 3D reconstruction was done using software to illustrate triple immunofluorescence in reactive astrocytes.

The selected brain areas from histological sections submitted to indirect immunoperoxidase (Ten-2-LI or ADGRL1-LI) were quantitatively and qualitatively analyzed in light microscope (Axiolab A1, Carl Zeiss, BW, Germany) coupled to a digital camera (AxioCam MRc5, Carl Zeiss, BW, Germany) associated to a microcomputer. For quantitative analyzes, five serial histological sections (480 μm equidistant) were selected from region CA3 of the hippocampus and cerebral cortex (primary somatosensory area) area of each animal (*n* = 4) of all experimental groups and then imaged using an imaging software (Zen2, Carl Zeiss, BW, Germany) under 10 × objective lens. Microphotographic imagens (TIFF format) were saved and analyzed using Image J software (Schneider et al., 2012), considering the immunoreactivity density (percentage of pixels/area). All layers (from I to VI) of the primary somatosensory area and CA3 (*lacunosum-moleculare, radiatum, lucidum, pyramidal, and oriens strata*) were considered for quantitative analyses. In addition, an image of each area selected for the study of the animal belonging to the 5-day group were converted to grades of gray, and then intensity, contrast, brightness, and threshold patterns were adjusted to the point where it was possible to clearly visualize reactive astrocytes Ten-2-LI or ADGRL1-LI. These parameters were performed for all analysis as previously described (King et al., 2002; Singh et al., 2012).

#### 2.5.7 Statistical analysis

Initially, the data from the immunoreactivity percentage of indirect immunoperoxidase reaction of region CA3 of the hippocampus and cerebral cortex were expressed as mean ± standard error (SEM) and submitted to Shapiro–Wilk test of normal distribution followed by analysis of variance (One-way ANOVA) and Tukey’s post-test, considering *p* < 0.05 as statistically significant. The data were processed using a statistical analysis program (GraphPad Prism 6, GraphPad Software, Inc., CA, USA).

### 2.6 RNA extraction and real-time PCR (qPCR)

Real-time PCR was performed to verify Ten-2, TCAP-2 and ADGRL1 gene expression changes in cerebral areas after SE induction using pilocarpine. For this, three animals (*n* = 3) for each group were anesthetized as previously described, positioned on a stereotaxic apparatus, then the cerebral tissue was exposed using surgical instruments. One brain slice of about 0.5 centimeters thick was removed and rinsed in DNase and RNase free ice-cold saline and then an additional trimming was conducted under surgical stereomicroscopy (Model MC A-199, DF Vasconcellos, RJ, Brazil) to collect a small region of interest (primary somatosensory area or region CA3 of the hippocampus). This fragment was placed into a specific centrifuge tube, weighed, added with 1.0 mL cold trizol (#15596026, Life Technologies, CA, USA) and immediately homogenized mechanically (985370EUR-04, Tissue-Tearor, Biospec Products, OK, USA) at 30,000 rpm for 40 s and incubated on ice for about 5 min. Next, 200 μL of chloroform (#0757, Biochemicals Life Science Research Products, OH, USA) were added, agitated for about 15 s, and maintained on ice for 10 min. After this step, the tubes were centrifuged (Mikro 220R, Hettech Zentrifugen, BW, Germany) at 12,000 × g for 15 min and the upper phase with total RNA was removed and transferred to a clean specific DNase and RNase free tube. Subsequently, 1.0 mL of 70% isopropyl alcohol was added and the tube with the total RNA sample was agitated for about 10 s and then a 700 μL aliquot of this solution was transferred to a purification column (RNeasy® Mini kit, #74106, Qiagen, TX, USA) and submitted to washes using specific buffers present in the commercial kit and centrifugations. Finally, the column eluant was transferred into the new and clean tube, where 100-μL of sterilized nuclease-free water (W4502, Sigma-Aldrich, MO, USA) were added and centrifuged at 8.000 × g for 1 min at 4 °C for total RNA resuspension. To avoid contamination with DNA, the samples of total RNA were treated with RNase-Free DNase Set (79,254, Qiagen, TX, USA) following the manufacturer’s instructions. The quality and quantity of the total RNA were measured using a spectrophotometer (NanoDrop 2000c UV–VIS, Thermo Fisher, MA, USA) and the RNA integrity was evaluated using the Bioanalyzer 2,100 (Bioanalyzer RNA Analysis, Agilent Technologies, CA, USA) and Aligent RNA 6000 Nano kit (Agilent Technologies, CA, USA). Subsequently, the quantity of total RNA for each sample was normalized and the cDNA synthesis was conducted using cDNA SuperScript III First-Strand Synthesis SuperMix (18,080,400, Thermo Fisher, MA, EUA) using 1 μg of total RNA. qPCR was performed using a Power SYBR Green I Master Mix (4,368,708, Thermo Fisher, MA, EUA) utilizing 5 μL of Power SYBR Green I Master Mix, 1 μL (300 nM) of forward and reverse primers to Ten-2 or TCAP-2, ADGRL1, GAPDH, neuron specific nuclear protein (NeuN) or RNA binding fox-1 homolog 3 (Rbfox3) (Table 1), 2 μL RNAse-free sterile H2O and 1 μL cDNA samples using a QuantStudio 12 K (Thermo Fisher, MA, EUA). The parameters for qPCR started with 10 min of incubation at 95° C, 40 cycles at 95° C for 15 s and 60° C for 1 min. The reactions were performed in triplicate. The set of primers was obtained using Primer Express software available on the QuantStudio 12 K equipment (Thermo Fisher, MA, USA). The data were normalized by GAPDH and expressed in relation to Rbfox3.

**Table 1:** Primer sets used in real-time technique, product size and accession number.

Gene	Primer pair (5′ to 3′)	Product size (base pairs)
Ten-2 (NM_011856.2)	(FWD) 5′-gatgtggacacagagacagaaggt-3′ (RVS) 5′-cattccccttatccacattcttagtg-3’	24 26
TCAP-2 (NM_011856.2)	(FWD) 5’-tgatggacggtgccttgaa-3′ (RVS) 5′-aaacagaagggacagcagcaa-3’	19 21
ADGRL1 (NM_181039.2)	(FWD) 5’-ccctacacactgcgtttcga-3′ (RVS) 5′-tggaggctgagcgcttgt-3’	20 18
GAPDH (NM_001289726.1)	(FWD) 5’-gctctctgctcctccctgttc-3′ (RVS) 5′-gaggctggcactgcacaa-3’	21 18
NeuN/ RBFOX3 (NC_000077.6)	(FWD) 5’-ccaccccccattccaact-3′ (RVS) 5′-ggcattttaacaagcgtttgc-3’	18 21

#### 2.6.1 qPCR and statistical analysis

For the qPCR data analyses, the dissociation curves were included in all amplifications to ensure primer specificity, specific origin of products and absence of DNA contamination. The Ct (threshold cycle) values, which constitute the number of cycles in the qPCR when a statistically significant amplification is detected for the first time in the reaction were exported for further analysis by the 2^ΔΔ^Ct method (Livak and Schmittgen, 2001; Schmittgen and Livak, 2008). The data were generated and presented according to the recommendations of the MIQE (Minimum Information for Publication of Quantitative Real-Time PCR Experiments) (Bustin et al., 2009).

The data from qPCR technique for samples of region CA3 of the hippocampus and cerebral cortex were expressed as mean ± standard error (SEM) and submitted to Shapiro–Wilk test of normal distribution followed by analysis of variance (One-way ANOVA) and Tukey’s post-test, considering *p* < 0.05 as statistically significant. The data were processed using a statistical analysis program (GraphPad Prism 6, GraphPad Software, Inc., CA, USA).

## 3 Results

### 3.1 Behavioral analysis of SE

Initially, the animals that received LiCl-pilocarpine administration (epileptic group – EG: EG2, EG5, EG14, EG35 and EG65 groups) showed mouth and facial movements, head nodding, forelimb clonus and jerking with forelimb clonus. After 15–40 min of Li-pilocarpine administration, the animals showed jerking, followed by falls with forelimb and hindlimb clonus (generalized motor seizures; Racine’s scale stage: 5; Racine, 1972). In addition, animals from EG35 and EG65 also showed tonic–clonic seizures (recurrent spontaneous seizures, chronic epilepsy) with an average duration of 20–30 s over the 30 and 60 days after SE induction, respectively. As described above, only animals classified as stage 5 on Racine’s scale were selected for this study. Animals from naïve (NAG) or pharmacological control (PCG) groups did not show any behavioral alterations compatible with Racine’s scale.

### 3.2 Immunohistochemistry technique control

Immunohistochemistry controls from immunoperoxidase staining or double immunofluorescence adopted in this study resulted in the complete absence of immunolabeling of neurons and astrocytes. No immunoreactivity was observed after primary or secondary antibody omissions. In addition, an adsorption test for Ten-2 was performed using different concentrations of control peptide (1:1; 1:0.1; 1:0.01; 1:0.005) and no immunolabeling was revealed (Tessarin et al., 2019).

### 3.3 Ten-2-LI in primary somatosensory area and hippocampus

**Figure 1:**
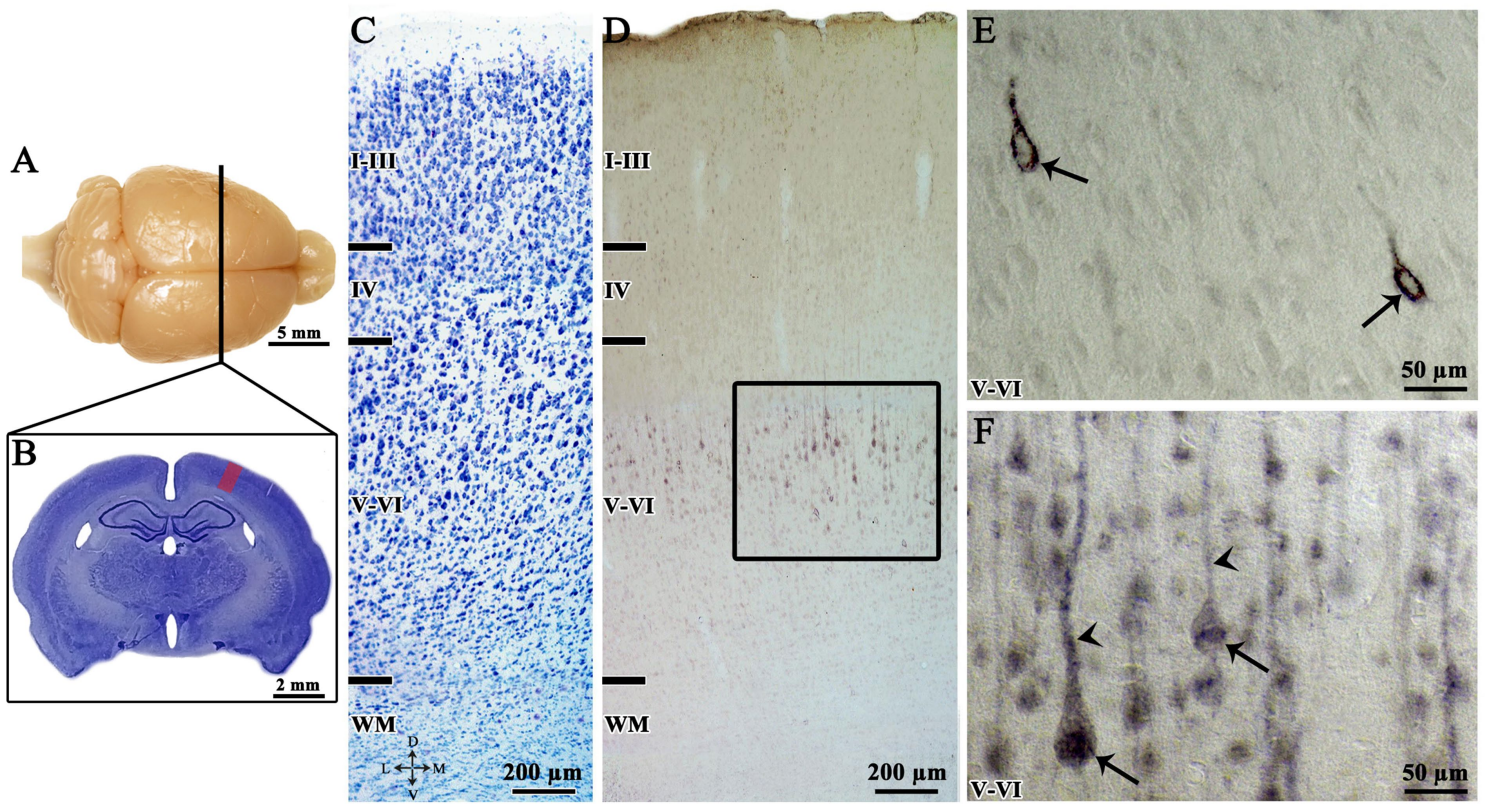
Anatomical dorsal view **(A)**, slice brain section stained with Nissl stain **(B,C)** and indirect immunoperoxidase **(D–F)** to analyze the cortical layers and Ten-2 immunolabeling pattern in histological section of adult rat primary somatosensory area of the NAG group. **(A,B)** Shows brain level (line in **A**, red area in **B**) of the analyzed primary somatosensory area. **(C)** Shows the supragranular (I–III), granular (IV), and infragranular (V, VI) layers. **(D)** Observe neurons Ten-2-LI predominantly distributed in infragranular layers, specifically in V layer. **(E)** (Enlargement of the square in **D**), note the presence of immunoreactivity to Ten-2 associated with the cell membrane (arrows). **(F)** (Enlargement of the square in **D**), immunoreactivity is distributed in cell cytosol (arrows) and associated with apical dendrite (arrowheads). I–III, supragranular layers; IV, granular layer; V, cerebral cortex layer V; V–VI, infragranular layers; D, dorsal; L, lateral; M, medial; Ten-2-LI, teneurin-2-like immunoreactive; V, ventral; WM, white matter.

The immunohistochemistry techniques used for assessment of Ten-2 in the cerebral cortex revealed discrete immunolabeling in pyramidal neurons (teneurin-2- like-immunoreactive, Ten-2-LI) located in layer V-VI of animals from NAG and PCG (Figure 1). Morphologically, two immuno-labeling patterns were observed in these neurons: (1) associated with the cell membrane surrounding the perikarya and (2) distributed in the cytoplasm of the perikarya (Figure 1).

**Figure 2:**
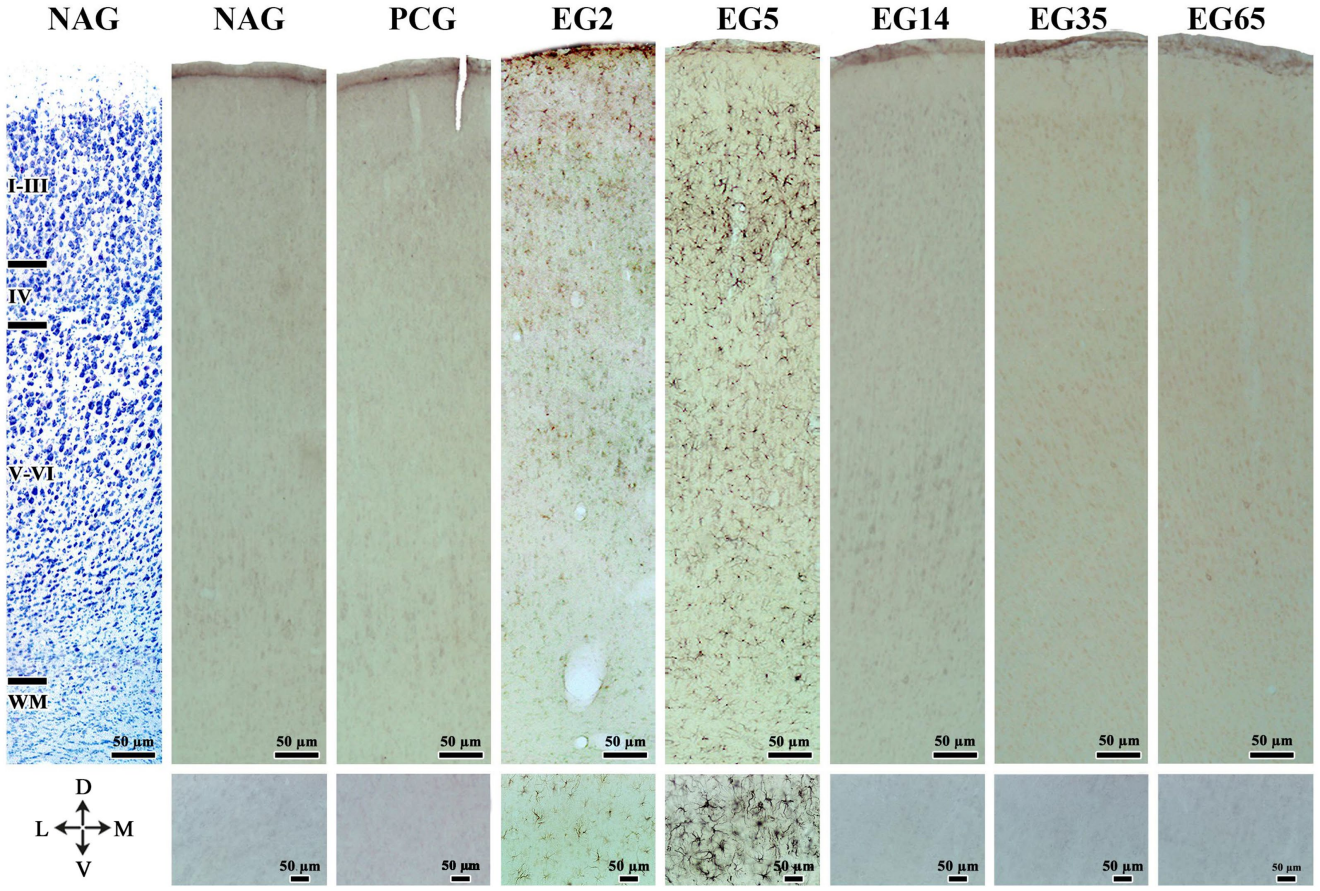
Nissl staining (NAG - left side) and temporal analysis of Ten-2-LI reactive astrocytes under light microscopy for all experimental groups in histological sections of the adult rat primary somatosensory area. Note a discrete immunoreactivity to Ten-2 in the EG2 group distributed in all layers of the cerebral cortex. In the EG5 group, observe the strong immunoreactivity to Ten-2 also distributed in all layers (mainly located in the supragranular layers) of the cerebral cortex. In other groups (NAG, PCG, EG14, EG35, and EG65) note the almost absence of Ten-2-LI reactive astrocytes. The small inferior figures of all groups show the enlargement of the supragranular area. I–III, supragranular layers; IV, granular layer; V, cerebral cortex layer V; V–VI, infragranular layers; D, dorsal; EG2, epilepsy group 2 days; EG5, epilepsy group 5 days; EG14, epilepsy group 14 days; EG35, epilepsy group 35 days; EG65, epilepsy group 65 days; L, lateral; M, medial; NAG, naïve group; PCG, pharmacological control group. Ten-2-LI, teneurin-2-like immunoreactive; V, ventral; WM, white matter.

**Figure 3:**
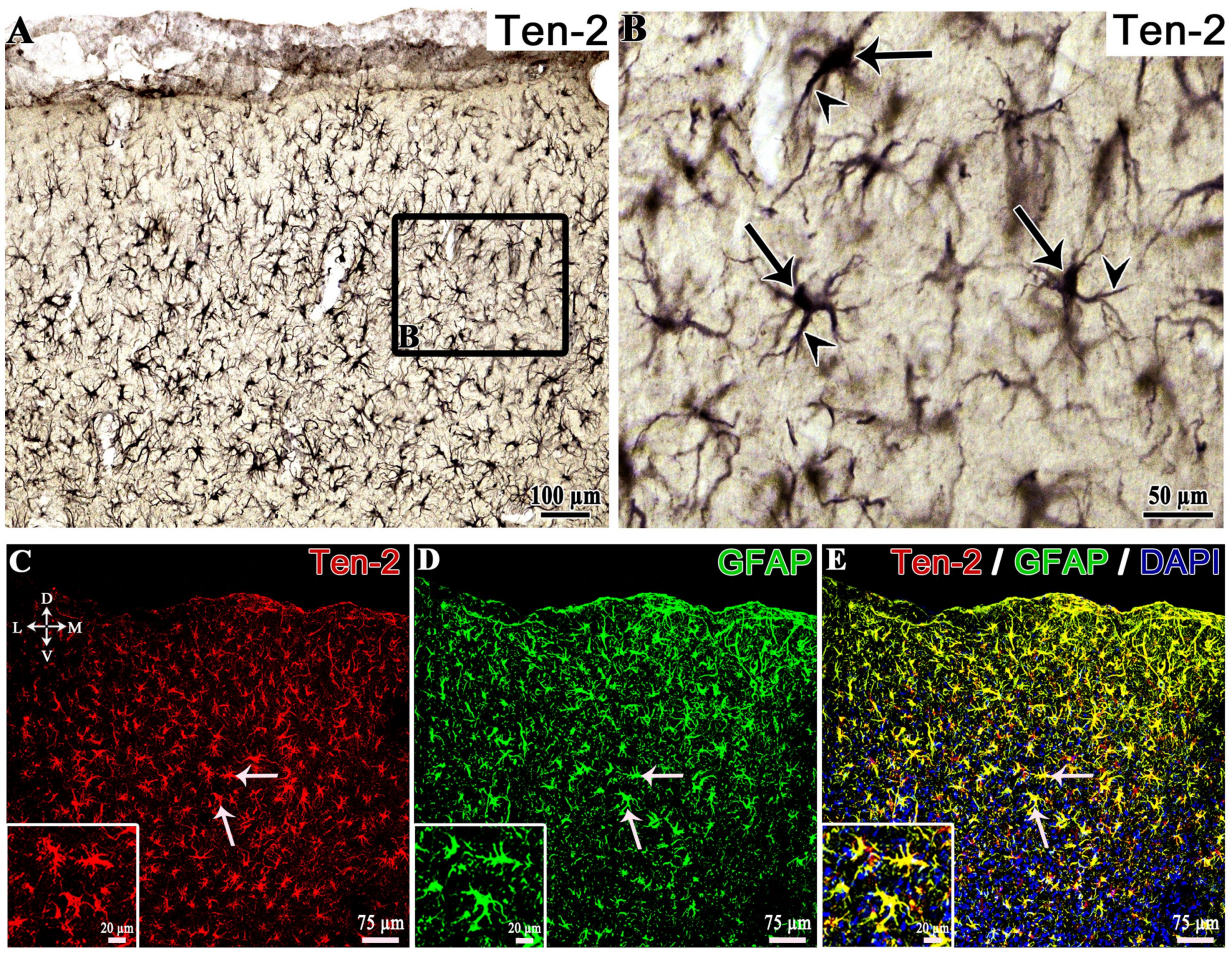
Ten-2-LI reactive astrocytes under light **(A,B)** and confocal microscopy **(C–E)** analysis in adult rat primary somatosensory area from an animal of EG5 group submitted to indirect immunoperoxidase **(A,B)** and double indirect immunofluorescence **(C–E)** stains. In panel **(A)**, note reactive astrocytes with strong immunoreaction to Ten-2. In panel **(B)** (enlargement of the black square in **A**) observe the presence of Ten-2 immunoreactivity associated to the cytosol of the cell body (arrows) and the cell extensions hypertrophied (arrowheads) and the cell extensions hypertrophied reactive astrocytes (arrowheads). In panel **(D)**, GFAP-LI reactive astrocytes (glial fibrillary acidic protein-like immunoreactive (GFAP-LI)). In panel **(E)**, double immunolabeling in reactive astrocytes (GFAP-LI/Ten-2-LI) with nuclear DAPI staining. The arrows show some double immunolabeling reactive astrocytes and the inset boxes exhibit enlargement of these cells. D, dorsal; GFAP, glial fibrillary acidic protein; L, lateral; M, medial; Ten-2, teneurin-2; V, ventral.

**Figure 4:**
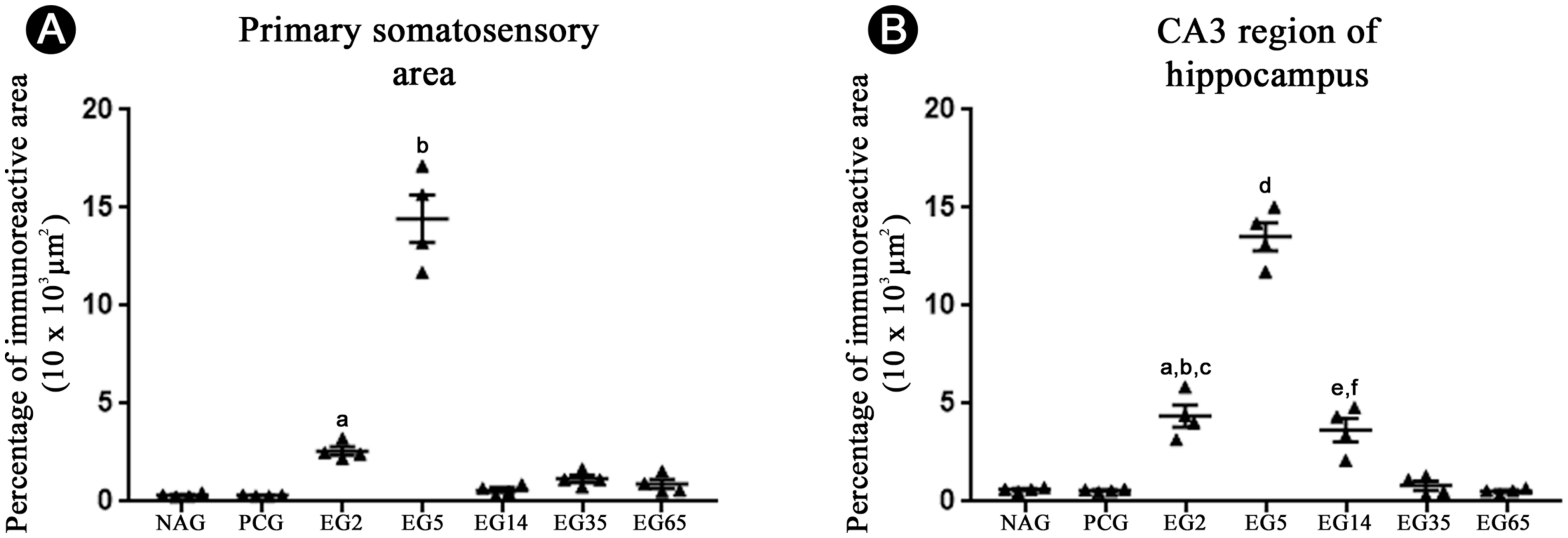
Temporal immunoreactivity density percentage (pixels/area) analysis to Ten-2 in the primary somatosensory area **(A)** and CA3 region of the hippocampus **(B)** in all experimental groups. Note a significant increase in EG2 and EG5 groups compared to other groups. Mean (± SEM) values from each experimental group were submitted to one-way ANOVA and Tukey’s *post hoc* test, considering *p* < 0.05 as significant. In panel **(A)**, a, EG2 vs. NAG or PCG (*p* < 0.05); b, EG5 vs. all other groups (*p* < 0.0001). In panel **(B)**, a, EG2 vs. NAG and PCG (*p* < 0. 0001); b, EG2 vs. EG35 (*p* < 0.001); c, EG2 vs. EG65 (*p* < 0.0001); d, EG5 vs. all other groups (*p* < 0. 0001); e, EG14 vs. NAG, PCG and EG65 (*p* < 0.001); f, EG14 vs. EG65 (*p* < 0.0001). CA3, cornu Ammonis 3 of the hippocampus; PCG, pharmacological control group; EG2, epilepsy group 2 days; EG5, epilepsy group 5 days; EG14, epilepsy group 14 days; EG35, epilepsy group 35 days; EG65, epilepsy group 65 days; NAG, naïve group.

The immunolabeling patterns changed substantially in animals submitted to SE (Figures 2, 3). Specifically, in EG5 animals, many reactive astrocytes showed strong immunoreactivity to Ten-2 (Figures 2, 3) distributed in all layers of the cerebral cortex (Figure 3). The same immuno-labeling pattern was evident in EG2; however, the immunoreactivity was not strong (Figure 2). Ten-2-LI reactive astrocyte profiles in EG2 and EG5 exhibited immunoreactivity distributed in the cytosol of the body and of cell processes hypertrophied (Figure 3). In other groups (EG14, EG35 and EG65), only a few Ten-2-LI reactive astrocyte profiles were detected in the cerebral cortex (Figure 2). Ten-2-LI neurons were also distributed amongst the Ten-2-LI astrocyte profiles in the cerebral cortex of the epileptic groups. It is also especially important to mention that astrocytes up-regulated glial fibrillary acidic protein (GFAP) after SE induction, characterizing an astrogliosis that is considered a hallmark of epilepsy (Figure 3). NAG and PCG revealed the lowest Ten-2-LI density (percentage of pixels/area), respectively, (77.41 μm^2^ ± 9.01 μm^2^) and (69.82 μm^2^ ± 7,36 μm^2^) (Figure 4). This immunoreactivity density came from constitutive Ten-2-LI cortical neurons. On the other hand, in EG2 (259.41 μm^2^ ± 21.80 μm^2^) an increase of immunoreactivity density from Ten-2-LI reactive astrocyte profiles was observed (Figure 4). Statistical differences between EG2 and NAG (*p* = 0.0446; Figure 4), and EG2 and control pharmacological group (*p* = 0.0429; Figure 4) occurred. The EG5 animals showed the highest immunoreactivity density (1,443.83 μm^2^ ± 121.87 μm^2^) among all experimental groups with significant statistical differences (*p* < 0.0001) (Figure 4). Interestingly, a decrease in immunoreactivity density in EG14 (59.98 μm^2^ ± 11.9 μm^2^), EG35 (117.46 μm^2^ ± 19.03 μm^2^) and EG65 (90.15 μm^2^ ± 23.19 μm^2^) was seen without statistical differences (Figure 4).

**Figure 5:**
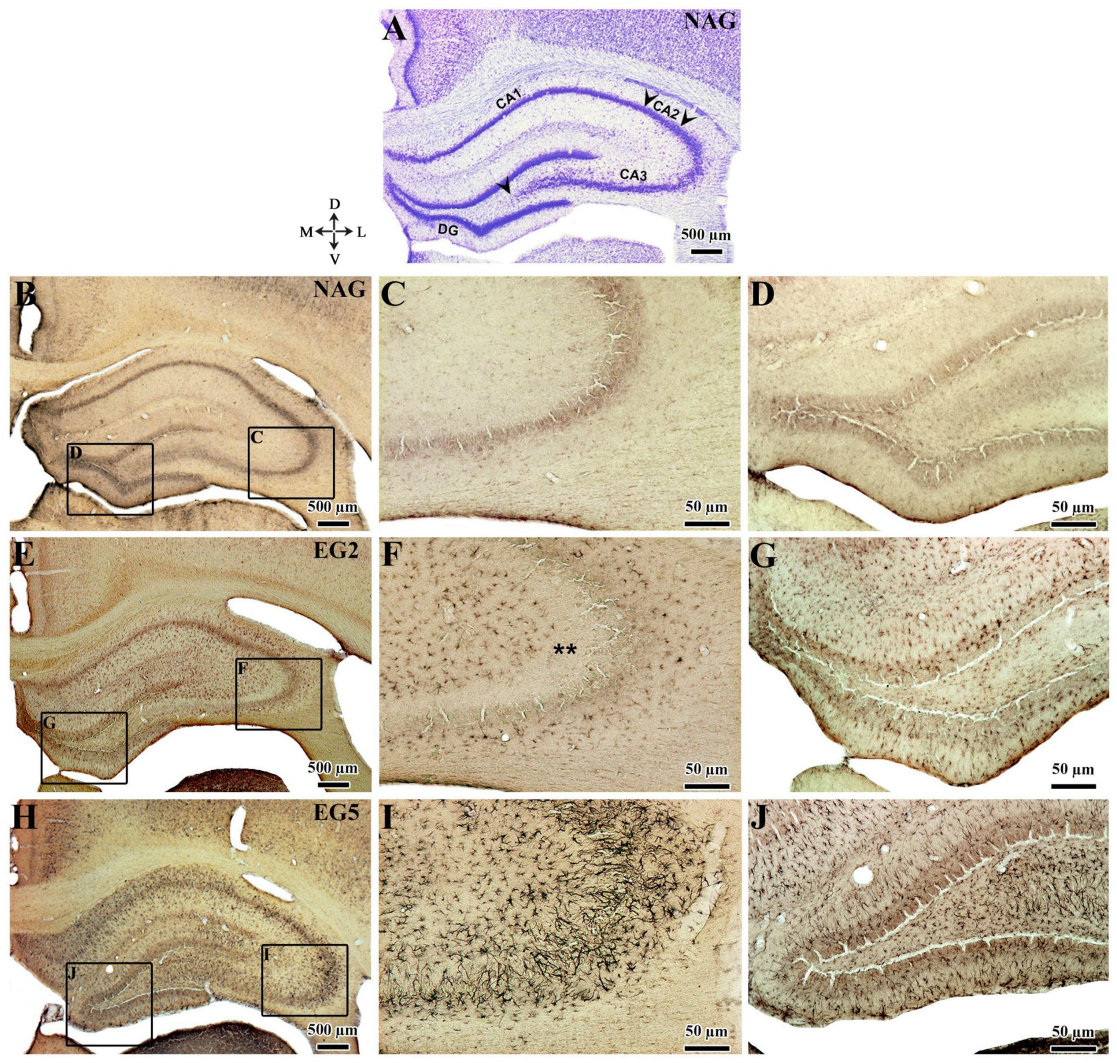
Nissl staining (NAG group); **(A)** and indirect immunoperoxidase to Ten-2 for naive **(B–D)**, EG2 **(E–G)**, and EG5 **(H–J)** groups from frontal histologic sections of the adult rat hippocampus analyzed under light microscopy. In panel **(A)**, note the subdivisions of the hippocampus (CA1, CA2, CA3, and dentate gyrus; delimited by arrowhead). In panel **(B)** observe the absence of immunoreactivity to Ten-2 in all subdivisions of the hippocampus as well as in CA3 **(C)** and dentate gyrus (D). In panel **(E–G)**, note the moderate intensity of Ten-2-LI astrocytic profiles in all subdivisions of the hippocampus **(E)**, subdivision CA3 **(F)**, and dentate gyrus **(G)** in EG2. In the stratum lucidum (**F**, asterisks) of the CA3 subdivision of the hippocampus, note the absence of Ten-2-LI astrocytic profiles. In panel **(H–J)**, observe the intense number of Ten-2-LI reactive astrocyte profiles in all subdivisions of the hippocampus **(H)**, subdivision CA3 **(I)**, and dentate gyrus **(J)**. The delimitations of the subdivisions of the hippocampal followed Swanson (1992). CA1, cornu Ammonis 1 of the hippocampus; CA2, cornu Ammonis 2 of the hippocampus; CA3, cornu Ammonis 3 of the hippocampus; D, dorsal; DG, dentate gyrus; L, lateral; M, medial; V, ventral.

In the hippocampus, immunohistochemical labeling of Ten-2 did not clearly reveal Ten-2-LI neurons in all experimental groups (Figure 5). In contrast, Ten-2-LI reactive astrocyte profiles were observed in the *stratum oriens*, *stratum pyramidale* and *stratum radiatum* of CA1, CA2 and CA3 regions in GE2 (Figure 5). However, in the *stratum lucidum* of CA3 region, no Ten-2-LI reactive astrocyte profiles were observed in EG2 (Figure 5, asterisks). In animals from EG5, the same immunolabeling patterns as in regions and layers of CA1, CA2 and CA3 were observed. Nevertheless, the immunoreactivity of Ten-2-LI reactive astrocyte profiles was strongest in this group (Figure 5). When present, *the stratum lucidum* of CA3 region also showed Ten-2 astrocyte profiles (Figure 5). In the dentate gyrus of the hippocampus, the laminar distribution of Ten-2-LI reactive astrocytes in the molecular, granule, polymorphic layers and hilus was similar between EG2 and EG5 (Figure 5). However, the immunochemical labeling intensity was strongest in EG5 (Figure 5). A lower immunolabelling intensity was noticed in animals from EG14, EG35 and EG65. Quantitative analysis of the CA3 region showed that NAG (62.37 μm^2^ ± 4.63 μm^2^) and PCG (57.76 μm^2^ ± 3.46 μm^2^) had the lowest immunoreactivity intensity (Figure 4). EG2 and EG5 showed a considerable increase of immunoreactivity density with mean values of 436.41 μm^2^ ± 55.73 μm^2^ and 1,351.76 μm^2^ ± 71.52 μm^2^, respectively; with a significant statistical difference among all remaining groups (*p* < 0.0001) (Figure 4). Elevated and persistent immunoreactivity density was still seen in animals from EG14 (365.46 μm^2^ ± 59.71 μm^2^), in contrast to when compared with the cerebral cortex (Figure 4). There were statistical differences between EG14 and NAG (*p* = 0.0007), control (*p* = 0.0008), EG35 (*p* = 0.0018) and EG65 (*p* = 0.0006) groups (Figure 4). Finally, EG35 (82.15 μm^2^ ± 23.36 μm^2^) and EG65 (54.40 μm^2^ ± 6.38 μm^2^) presented a decrease of immunoreactivity density, albeit without statistical difference.

**Figure 6:**
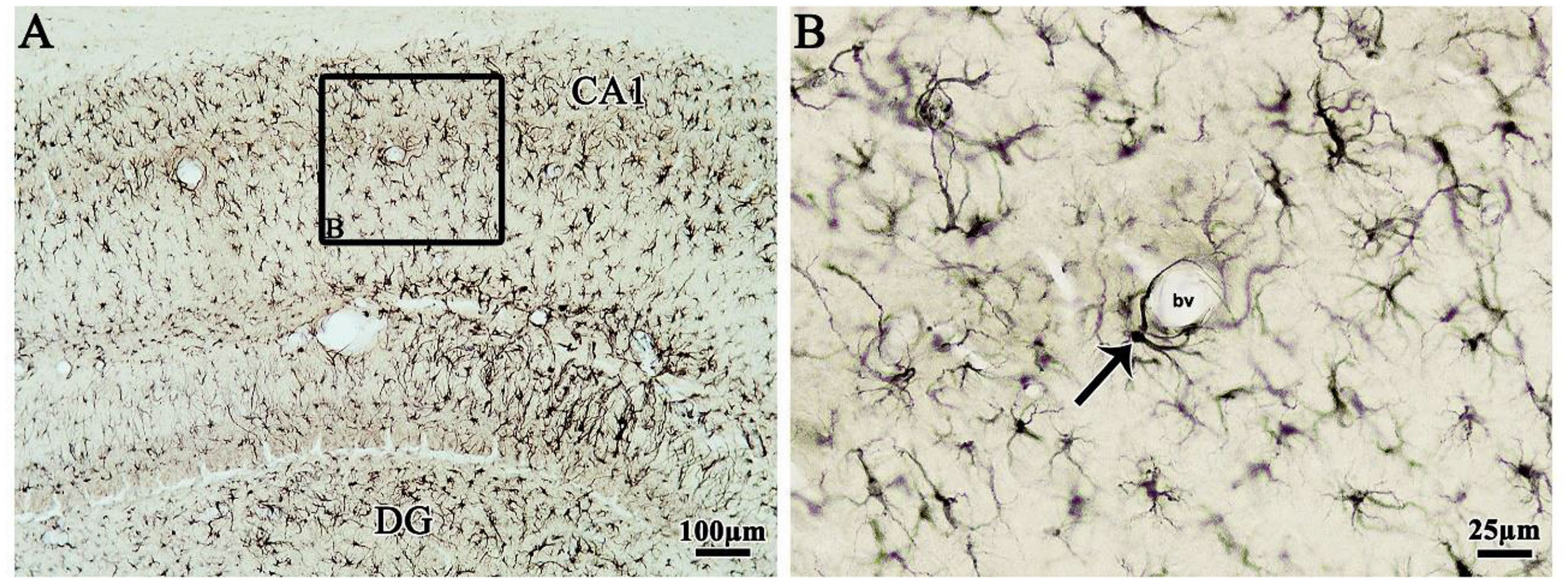
Indirect immunoperoxidase to Ten-2 staining from an animal of EG5 group in the frontal histologic section of the adult rat hippocampus analyzed under light microscopy showing the CA1 and DG subdivisions. **(A)** Observe the Ten-2-LI astrocyte profiles in the CA1 and DG subdivisions. **(B)** [Enlargement of black square observed in panel **(A)**], note also Ten-2-LI reactive astrocyte profiles in the CA1 subdivision and a specific Ten-2-LI astrocyte profile with its cell extension in contact with the blood vessel (arrow). bv, blood vessel; CA1, cornu Ammonis 1 of the hippocampus; D, dorsal; DG, dentate gyrus; L, lateral; M, medial; V, ventral.

Interestingly, the presence of Ten-2-LI reactive astrocyte profiles with their cell extension encircling blood vessels was quite common in the cerebral cortex as well as in the hippocampus (Figure 6).

### 3.4 ADGRL1-LI in primary somatosensory area and hippocampus

**Figure 7:**
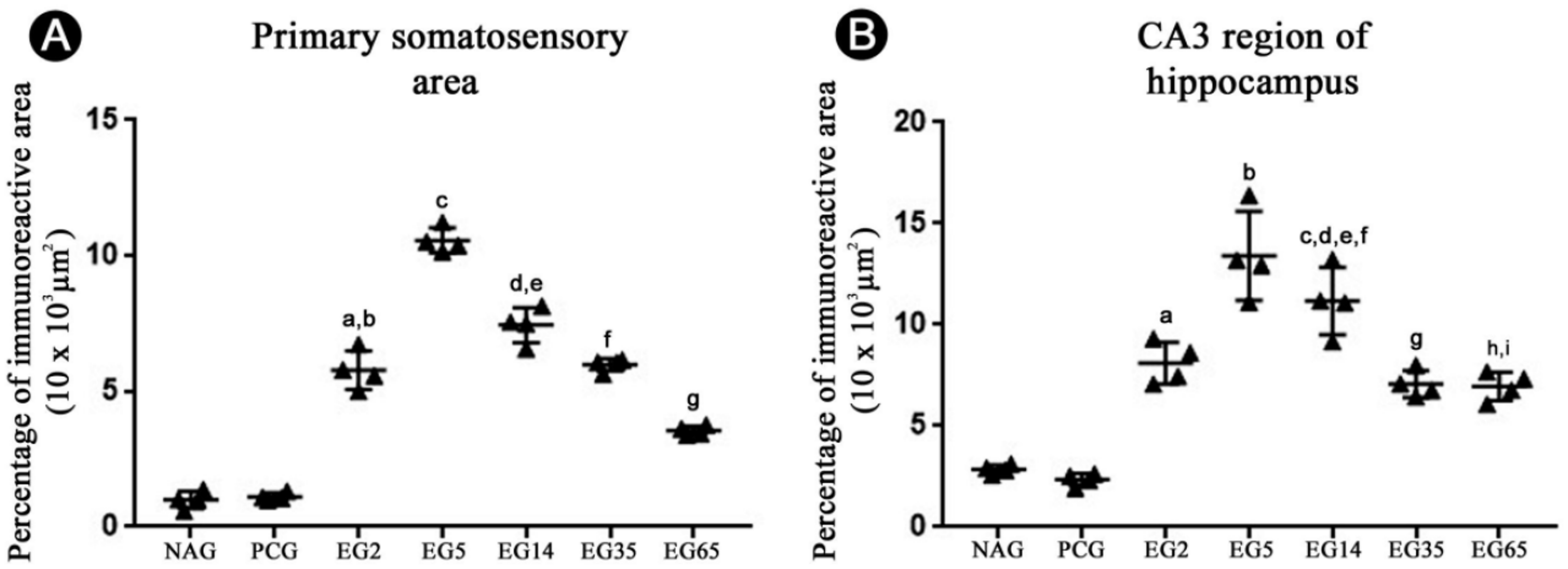
Temporal immunoreactivity density percentage (pixels/area) analysis to ADGRL1-LI in the primary somatosensory area **(A)** and CA3 region of the hippocampus **(B)** in all experimental groups. Note a significant increase, principally in EG5 and EG14 compared to other groups. Mean (± SEM) values from each experimental group were submitted to one-way ANOVA and Tukey’s post hoc test, considering *p* < 0.05 as significant. In panel **(A)**, a, EG2 vs. EG14 (*p* < 0.001); b, EG5 vs. NAG, PCG and EG65 (*p* < 0.0001); c, EG5 vs. all other groups (*p* < 0.0001); d, EG14 vs. NAG, PCG and EG65 (*p* < 0.0001); e, EG14 vs. EG35 (*p* < 0.01); f, EG35 vs. NAG and PCG (*p* < 0.0001); g, EG65 vs. NAG and PCG (*p* < 0.0001). In panel **(B)**, a, EG2 vs. NAG and PCG (*p* < 0.0001); b, EG5 vs. NAG, PCG, EG2, EG35 and EG65 (*p* < 0.0001); c, EG14 vs. NAG, PCG and EG60 (*p* < 0.0001); d, EG14 vs. EG2 (*p* < 0.05); e, EG14 vs. EG35 (*p* < 0.01); f, EG14 vs. EG65 (*p* < 0.001); g, EG35 vs. NAG, PCG (*p* < 0.001); h, EG65 vs. NAG (*p* < 0.01); EG65 vs. NAG (*p* < 0.001). ADGRL1, adhesion G protein-coupled receptor L1; CA3, cornu mmonis 3 of the hippocampus; PCG, pharmacological control group; EG2, epilepsy group 2 days; EG5, epilepsy group 5 days; EG14, epilepsy group 14 days; EG35, epilepsy group 35 days; EG65, epilepsy group 65 days, NAG, naïve group.

ADGRL1-LI immunoreactivity was present discretely in astrocytes homogenously distributed in all layers of the primary somatosensory area in NAG and PCG. This immunoreactivity increased from EG2 (51.60 μm^2^ ± 3.20 μm^2^) to EG5 (225.7 μm^2^ ± 4.95 μm^2^), persisting in the superficial layers (I-III) of the cerebral cortex in EG14 (96.14 μm^2^ ± 4.20 μm^2^), EG35 (66.74 μm^2^ ± 1.9 μm^2^) and EG65 (76 μm^2^ ± 1.80 μm^2^) (Figure 7). All epileptic groups showed significant differences (*p* < 0.0001) when compared with NAG and PCG (Figure 7). There was no statistical difference between NAG and PCG (Figure 7).

**Figure 8:**
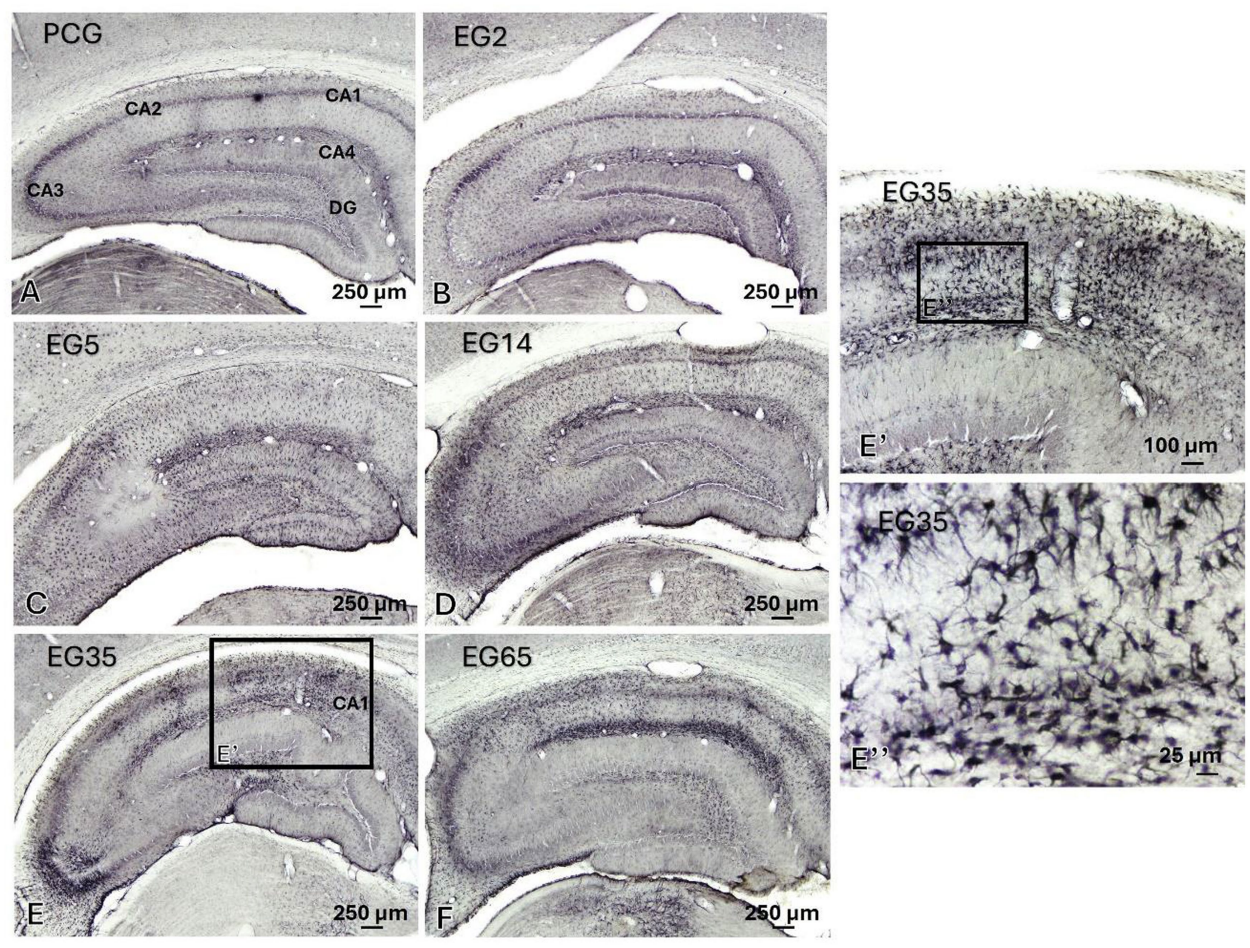
Indirect immunoperoxidase to ADGRL1-LI for PCG and all EG groups from frontal histologic sections of the adult rat hippocampus analyzed under light microscopy **(A–F)**. In panel **(A)**, note the subdivisions of the hippocampus (CA1, CA2, CA3 and dentate gyrus). In panel **(E’)** observe ADGRL1-LI reactive astrocytes in all layers of CA1 from EG35 and E” shows these cells distributed in stratum radiatum and stratum-lacunosum-moleculare. CA1, cornu Ammonis 1 of the hippocampus; CA2, cornu Ammonis 2 of the hippocampus; CA3, cornu Ammonis 3 of the hippocampus; D, dorsal; DG, dentate gyrus; L, lateral; M, medial; V, ventral.

ADGRL1-LI was constitutively present in astrocytes homogenously distributed in all layers of CA3 region of the hippocampus in NAG (11.94 μm^2^ ± 0.5 μm^2^) and PCG (12 μm^2^ ± 0.8 μm^2^) (Figure 7). This percentage of immunoreactivity intensity was increased in EG2 and principally in EG5 (235 μm^2^ ± 20 μm^2^) and EG14 (145.3 μm^2^ ± 10.8 μm^2^) and persisted in remaining epileptic groups (EG35, 42.8 μm^2^ ± 2 μm^2^; EG65, 82.70 μm^2^ ± 4.2 μm^2^) (Figure 7). ADGRL1-LI in the hippocampus from EG14, EG35 and EG65 was heterogeneously distributed in the CA and DG regions as well as into cell layers (Figure 8). In addition, certain regional differences occurred among animals from specific epileptic groups. The immunoreactivity was situated in all layers of CA1 in EG35 and EG65 (Figure 8). Quantitative analysis showed that EG5 and EG14 groups exhibited significant differences in relation to the remaining groups (p < 0.0001), and all epileptic groups showed significant differences (p < 0.0001) when compared with NAG and PCG (Figure 7). There was no statistical difference between NAG and PCG groups (Figure 7).

### 3.5 Colocalization of Ten-2- and ADGRL1-LI in reactive astrocytes (GFAP)

**Figure 9:**
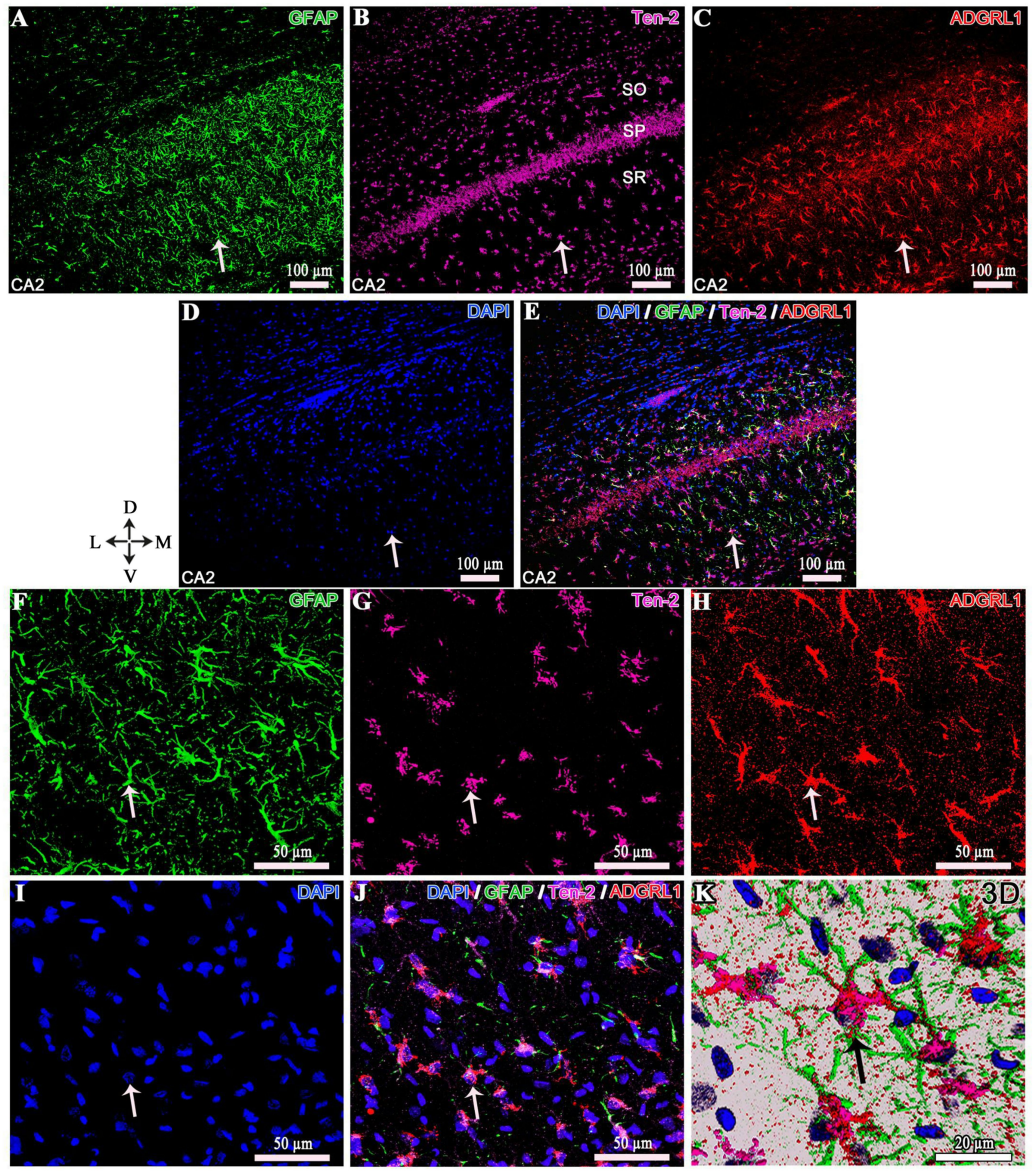
Triple immunofluorescence of the hippocampus from EG5 epileptic group. Low magnification **(A–E)** and high magnification **(F–J)** of reactive astrocytes, exhibiting GFAP, Ten-2-LI, ADGRL1-LI and DAPI labeling under confocal microscopy. In panel **(K)**, a 3D reconstruction of a reactive astrocyte with triple immunolabeling is shown, identified by the arrows. ADGRL1, adhesion G protein-coupled receptor L1; CA2, cornu Ammonis 2 of the hippocampus; D, dorsal; GFAP, glial fibrillary acidic protein; L, lateral; M, medial; SO*, stratum oriens*; SP, *stratum pyramidale*; SR, *stratum radiatum*; Ten-2, teneurin-2; V, ventral.

Simultaneous immunolabeling using three specific antibodies (GFAP, Ten-2 and ADGRL1) showed that a substantial number of GFAP positive reactive astrocytes in the hippocampus and cerebral cortex exhibited colocalization of Ten-2 and ADGRL1 in animals of the epilepsy groups (Figure 9). The antibody for Ten-2 (R&D system) used in this assay was different from the one used in immunoperoxidase analysis (Santa Cruz Biotechnology), since we decided to adopt three distinct primary antibodies raised in different animal species to permit a correct triple indirect immunofluorescence analysis. The antibody used in this assay to Ten-2 showed the immunopositivity concentrated in the cell body of reactive astrocytes and rarely in the cell extensions (Figure 9). The ADGRL1 and GFAP antibodies labeled all parts of the reactive astrocytes, including all cell extensions resulting in immunolabeling of the cell body, cell extensions and rich fine punctuate or granulated profiles of the cell extensions (Figure 9).

### 3.6 Ten-2-LI reactive astrocyte profiles and neuronal degeneration

**Figure 10:**
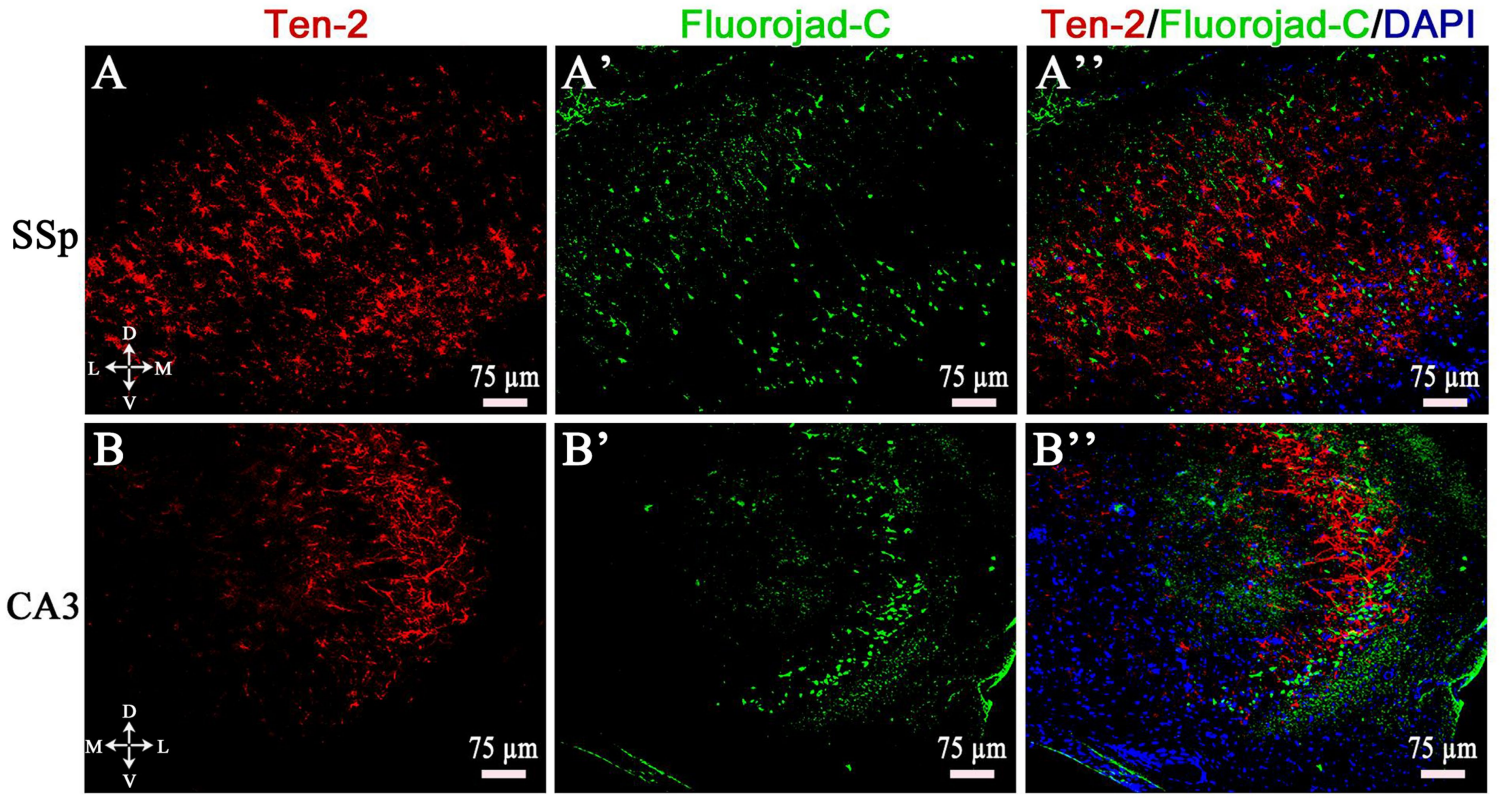
Double labeling of Ten-2-LI/ Fluoro jade-C in the primary somatosensory area **(A–A”)** and CA3 **(B–B”)** in a histologic section from an animal of EG5 group. Note that Ten-2-LI reactive astrocytes coincide with neurons stained with Fluoro-Jade C staining. CA3, cornu Ammonis 3 of the hippocampus; D, dorsal EG5, epilepsy group 5 days; L, lateral; M, medial; SSp, primary somatosensory area; Ten-2, teneurin-2; V, ventral.

Fluoro-Jade C staining, combined with immunofluorescence, revealed the presence of Ten-2-LI reactive astrocyte profiles in areas with neuronal degeneration in the cerebral cortex and CA3 of the hippocampus after SE induction, mainly in EG2 and EG5 (Figure 10). Specifically in the cerebral cortex, intense neuronal degeneration was also present in animals from EG14, EG35 and EG65 associated with a few Ten-2-LI-reactive astrocyte profiles. Additional CNS regions exhibited strong Ten-2-LI reactive astrocytes, mainly in the CA1, CA2 and dentate gyrus of the hippocampus, lateral nucleus and anterodorsal nuclei of the thalamus, central nucleus of the amygdala, piriform and entorhinal cortices, besides other areas (Supplementary Figures S1–S3). All those regions also showed consistent colocalization with neuronal degeneration (Supplementary Figure S3).

### 3.7 Ten-2, TCAP-2 and ADGRL1 gene expression

**Figure 11:**
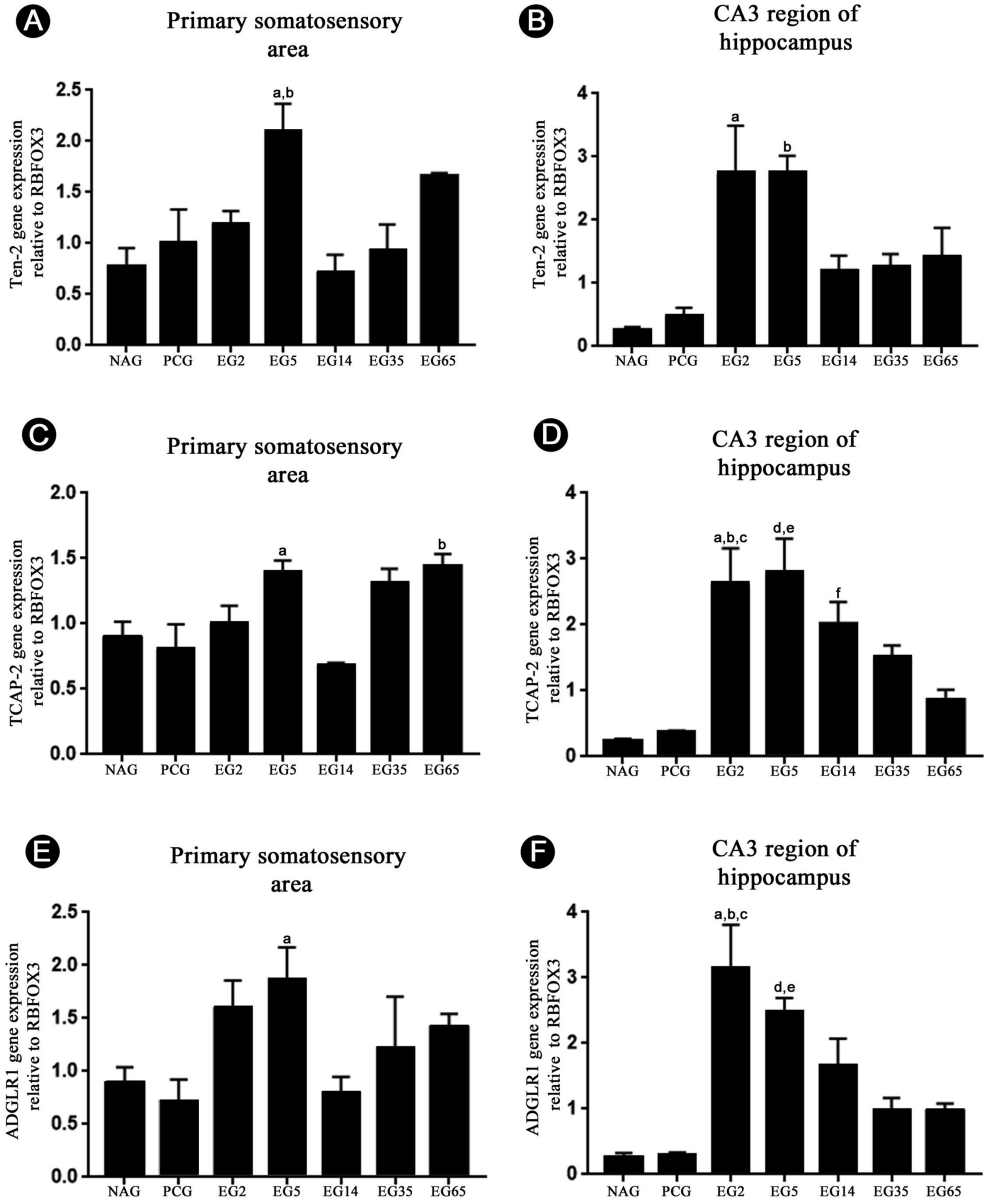
Temporal quantitative genic expression analysis to Ten-2 **(A,B)**, TCAP-2 **(C,D)**, and ADGRL1 **(E,F)** from primary somatosensory area and hippocampus samples (*n* = 3, four animals per experimental group). Note the increase of gene expression to Ten-2 **(A,B)**, TCAP-2 **(C,D)**, and ADGRL1 **(E,F)** especially in the EG2 and EG5 groups. Mean (± SEM) values from each experimental group normalized in relation to GADPH and expressed in relation to NeuN (RBFOX3) submitted to one-way ANOVA and Tukey’s post hoc test, considering *p* < 0.05 as significant. In panel **(A)**, a EG5 vs. PCG and EG35 (*p* < 0.05); b EG5 vs. NAG and EG14 (*p* < 0.01). In panel **(B)**, a EG2 vs. NAG and PCG (*p* < 0.01); b EG5 vs. NAG and PCG (*p* < 0.01). In panel **(C)**, a EG5 vs. EG14 (*p* < 0.05); b EG65 vs. EG14 (*p* < 0.01). In panel **(D)**, a EG2 vs. EG65 (p < 0.05); b EG2 vs. NAG (*p* < 0.001); c EG2 vs. PCG (*p* < 0.01); d EG5 vs. EG65 (*p* < 0.05); e EG5 vs. NAG and PCG (*p* < 0.001); f EG14 vs. NAG and PCG (*p* < 0.05). In panel **(E)**, a EG5 vs. NAG and PCG (*p* < 0.05). In panel **(F)**, a EG2 vs. EG14 (*p* < 0.05); b EG2 vs. NAG and PCG (*p* < 0.0001); c EG2 vs. EG35 and EG6 (*p* < 0.001); d EG5 vs. NAG and PCG (*p* < 0.05); e EG5 vs. NAG and PCG (*p* < 0.001). ADGRL1, adhesion G protein-coupled receptors L1; PCG, control group; EG2, epilepsy group 2 days; EG5, epilepsy group 5 days; EG14, epilepsy group 14 days; EG35, epilepsy group 35 days; EG65, epilepsy group 65 days; GHPDH, glyceraldehyde 3-phosphate dehydrogenase;; NeuN, neuron specific nuclear protein; RBFOX3, RNA binding Fox-1 homolog 3; TCAP-2, teneurin C-terminal-associated peptide 2; Ten-2, teneurin-2.

The qRT-PCR analysis was used to confirm Ten-2 and ADGRL1 expression in the cerebral cortex and hippocampus, as well as to verify if TCAP-2 is also expressed in the brain after SE, since there is no available and specific antibody to TCAP-2. In addition, this technique was performed to check the possible changes in mRNA of Ten-2, ADGRL1 and TCAP-2 after SE. Initially, the gene expression of Ten-2, TCAP-2 and ADGRL1 was seen in all experimental groups (Figures 11A–D). Cerebral cortex analysis revealed up-regulation of Ten-2 gene in animals from EG5 (2,084 ± 0,314, Figure 11A). Statistically, there were significant differences among EG5 and NAG (0.775 ± 0.199; *p* = 0.0051), PCG (0.988 ± 0.385; *p* = 0.0269), EG14 (0.703 ± 0.207; *p* = 0.0029) and EG35 (0.921 ± 0.298; *p* = 0.0161) (Figure 11A). However, the gene expression of TCAP-2 was slightly different in relation to Ten-2. TCAP-2 gene up-regulation was observed in animals from EG5 (1.258 ± 0.163), EG35 (1.223 ± 0.135) and EG65 (1.424 ± 0.125) (Figure 11C). The NAG (0.955 ± 0.126), PCG (0.924 ± 0.236) and EG14 (0.638 ± 0.043) showed the lowest relative quantity. Statistical differences were evidenced only between EG5 and EG14 (*p* = 0.0347) and between EG14 and EG65 (*p* = 0.0018) (Figure 11C).

In the CA3 region of hippocampus, the relative gene expression of Ten-2 for the NAG (0.269 ± 0.042) and PCG (0.468 ± 0.158) groups showed the lowest means (Figure 11B). However, the highest up-regulation of Ten-2 gene was evident in EG2 (2.292 ± 0.895) and EG5 (2.719 ± 0.335) (Figure 11B). There was a down-regulation of Ten-2 in EG14 (1.094 ± 0.271), EG35 (1.143 ± 0.314) and EG65 (1.408 ± 0.524) in relation to EG2 and EG5 (Figure 11B). Statistical differences were present between EG2 and NAG (*p* = 0.0022) and PCG (*p* = 0.0057). Significant differences were also detected between EG5 and NAG (*p* = 0.0019) and PCG (*p* = 0.0050) (Figure 11B). TCAP-2 gene expression was lowest in the NAG (0.241 ± 0.034) and PCG (0.378 ± 0.023) (Figure 11D). In both the EG2 (2.589 ± 0,638) and EG5 (2,732 ± 0,644) groups, an up-regulation of TCAP-2 gene occurred (Figure 11D). EG14 (2.019 ± 0.364), EG35 (1.480 ± 0.233) and EG65 (0.854 ± 1.180) showed a significant downregulation to TCAP-2 gene (Figure 11D). Significant statistical differences were present between EG2 and NAG (*p* = 0.0008), PCG (p = 0.0018) and EG65 (*p* = 0.0208) (Figure 11D) and EG5 with NAG (*p* = 0.0004), PCG (p = 0.0008) and EG65 (*p* = 0.0101) (Figure 11D). Finally, EG14 also showed a significant increase when compared with NAG (*p* = 0.0167) and PCG (*p* = 0.0328) (Figure 11D).

ADGRL1 gene expression was altered in the cerebral cortex and CA3 region of hippocampus after epilepsy induction (Figures 11E,F). Using cerebral cortex samples, the highest up-regulation of ADGRL1 gene was evident in EG2 (2.292 ± 0.895) and EG5 (2.719 ± 0.335) (Figure 11E). NAG (0.750 ± 0.116), PCG (0.711 ± 0.235) and EG14 (0.783 ± 0.183) showed the lowest expression of ADGRL1 gene (Figure 11E). In EG35 (1.245 ± 0.526) and EG65 (1.478 ± 0.098) groups, slight increases of ADGRL1 gene expression were noticed. (Figure 11E). Statistical differences were only observed between EG5 with NAG (*p* = 0.0438) and PCG (*p* = 0.0338) (Figure 11E). In the hippocampus, the highest gene expression to ADGRL1 was seen in EG2 (3.144 ± 0.739) with statistical differences with NAG (0.221 ± 0,064), PCG (0.315 ± 0,039; *p* < 0.0001), EG14 (1.899 ± 0.654; *p* = 0.0369), EG35 (1.154 ± 0.099; *p* = 0.0008) and EG65 (1.209 ± 0.048; *p* = 0.0007) (Figure 11F). Analyses using samples from EG5 (2.473 ± 0.241) showed slight decreases of ADGRL1 gene expression when compared with EG2 (Figure 11F). Statistical differences were evident between EG5 and NAG (*p* = 0.0006), control (*p* = 0.0007), EG35 (*p* = 0.0366) and EG65 (*p* = 0.0318) (Figure 11F). EG14 (1.659 ± 0.459), EG35 (0.987 ± 0.199) and EG65 (0.960 ± 0.134) showed a decrease of ADGRL1 gene expression in relation to EG2 and EG5 (Figure 11F). NAG (0.261 ± 0.070) and PCG (0.290 ± 0.048) showed the lowest gene expressions (Figure 11F).

## 4 Discussion

This study is the first to show that Ten-2/TCAP-2 system and its main cognate receptor, ADGRL1, are upregulated in reactive astrocytes in the primary somatosensory area and CA3 region of hippocampus in a LiCl-pilocarpine rat model of epilepsy, using immunohistochemistry and gene expression analysis. This study indicates that a teneurin/TCAP-ADGRL coupling likely plays a role in CNS with seizure disorders or epilepsy, predominantly in areas with neuronal degeneration.

### 4.1 Technical considerations

The epilepsy model adopted in the present study has been widely used to evaluate the *status epilepticus* (SE), as well as recurrent spontaneous seizures (chronic epilepsy) and their neuropathological alterations (Turski et al., 1983; Cavalheiro, 1995; Scorza et al., 2009; Ahmed Juvale and Che Has, 2020; Lévesque et al., 2021). The protocol implemented in this study combined LiCl and pilocarpine drugs, permitting the use of a lower dose of pilocarpine (40 mg/kg), different from the use of high doses when only pilocarpine is used (300–400 mg/kg) (Ahmed Juvale and Che Has, 2020; Lévesque et al., 2021). The LiCl-pilocarpine protocol reduced mortality rates (40–60%) in the first two days after SE. A few studies have also adopted repeated doses of pilocarpine (10 mg/Kg) to obtain SE, after LiCl treatment, and significantly decreased mortality rates to 10% (Ahmed Juvale and Che Has, 2020; Glien et al., 2001). Nevertheless, we decided to use one dose of 40 mg/kg of pilocarpine and selected only the animals with SE classified as stage 5 on Racine scale (Racine, 1972), avoiding possible additional variables, such as seizure-resistant animals or different pilocarpine doses used for SE induction.

The primary antibodies chosen to identify Ten-2 or ADGRL1 in reactive astrocytes by indirect immunoperoxidase for quantification showed a divergent immunoreactivity pattern when compared to other available commercial antibodies, evaluated in our laboratory. Even though all the antibodies showed acceptable immunolabeling of neurons, the Ten-2 antibodies tested in the immunoperoxidase analysis showed different patterns of immunoreactivity in reactive astrocytes, despite both using immunogen of the N-terminal cytoplasmatic domain of human teneurin-2. The Ten-2 antibody adopted in the immunoperoxidase analysis showed clear immunolabeling in all parts of the reactive astrocytes and less immunolabeling of neurons, whereas for other antibodies, this immunolabeling was more present along the cell extension or in the cell body of reactive astrocytes, impairing an adequate cell identification and quantification of these cells. Another point is that some commercially available antibodies for Ten-2 were discontinued, not permitting to compare immunolabeling efficiency among previously published studies. The Ten-2 antibody adopted in the indirect immunoperoxidase assay was used in an earlier study that performed absorption test and additional immunohistochemistry control, permitting us to confirm its adequate specificity in histologic sections from the rat CNS (Tessarin et al., 2019). Commercially available ADGRL1 antibodies also showed different efficacy, some antibodies showed immuno-labeling almost absent in neurons and glia cells, or the immunolabeling was so weak that it was not possible to clearly identify which cell exhibited immunoreactivity. The ADGRL1 antibody adopted in this study clearly showed immunolabeling of astrocytes and neurons in control groups, as well as neurons and reactive astrocytes in animals from SE groups.

Triple indirect immunofluorescence was important to confirm the colocalization of Ten-2 and ADGRL1 in the same reactive astrocytes (GFAP immunopositive). In this technique, we selected a combination of three primary antibodies raised in distinct animal species to avoid cross-reactivity of the secondary antibodies. However, Ten-2 antibody used in this triple immunolabeling showed immunoreactivity restricted in the cell body, different from the one used in the immunoperoxidase analysis.

Despite the gene expression analysis definitively showing Ten-2, TCAP-2 and ADGRL1 upregulation, these alterations were only statistically significant in certain groups. Some discrepancies between gene expression and immunohistochemistry data found in this study can potentially be explained because it is not possible to separate the gene expression profile from the neurons and reactive astrocytes using real time-PCR, based on the methodology used. The epileptic groups showed considerable degeneration of neurons confirmed by Fluoro-Jade C staining; therefore, the increase of Ten-2, TCAP-2 and ADGRL1 gene expression in these groups must be due to up-regulation of the genes from reactive astrocytes. There is an additional methodological limitation concerning gene expression data, as a considerable number of neurons degenerate after SE induced by pilocarpine, as shown in earlier studies and in the present study (Wang et al., 2008; Scholl et al., 2013). To help account for this, Ten-2, TCAP-2 and ADGRL1 gene expressions were first normalized using housekeeping genes, and data was expressed in relation to NeuN or Rbfox3 gene expression, a neuronal marker. This analysis allowed us to present more accurate data, considering that gene expression from neurons was reduced due to substantial neurodegeneration.

Another reasonable explanation for the slight discrepancy between immunohistochemistry and gene expression analysis is that the former permits an accurate quantification of what kind of cell exhibits immunoreactivity; while the latter encompasses a fraction of different cells from primary somatosensory area or CA3 hippocampus, which sometimes also include other adjacent areas besides the one analyzed in the immunohistochemistry quantifications. Thus, this did not permit us to observe accurate regional differences as noticed in the immunohistochemistry techniques.

### 4.2 Reactive astrocytes and Ten-2/TCAP-2/ADGRL1

Several studies have shown that astrocytes take part in many kinds of damage in the CNS such as infections, traumatic brain injury, stroke, neurodegenerative disorders, epilepsy, and others (Sofroniew, 2014; Burda et al., 2016; Liu and Chopp, 2016; Magaki et al., 2018; Verhoog et al., 2020; Verkhratsky et al., 2023). Under conditions in which alterations in homeostasis occur, the astrocytes up-regulate glial fibrillary acidic protein (GFAP), vimentin, other types of proteins and various cell factors that distinguish the reactive astrocytes (astrogliosis) (Sofroniew, 2014; Magaki et al., 2018; Todd and Hardingham, 2020). In the present study, using immunohistochemical methods, the animals from SE groups showed an increase in GFAP immunopositive hypertrophied astrocytes in the cerebral cortex and hippocampus proving the existence of reactive astrocytes. GFAP reactive astrocytes persisted throughout experimental time study in the primary somatosensory area and hippocampus, as well as in other regions of the CNS where substantial neuronal damage is present.

Previous studies indicated the presence of reactive astrocytes after pathologies of the CNS, such as in the early and late phases of epilepsy, corroborating the findings of the present study (Sofroniew and Vinters, 2010; García-García et al., 2017; Vizuete et al., 2017; Vizuete et al., 2018). The immunohistochemical data found a transient (EG2 and EG5) and strong immunoreactivity to Ten-2 and a more persistent immunoreactivity to ADGRL1 (EG2-EG65) in typical reactive astrocytes distributed in the cerebral cortex and hippocampus. A prior study also showed the presence of Ten-2-LI or ADGRL1 in reactive astrocytes after a needle-insertion lesion model in the cerebral cortex of adult rats (Tessarin et al., 2019). Furthermore, an increase of Ten2-LI or ADGRL1-LI in neurons or in microglial cells and oligodendrocytes did not occur in the present study. Thus, reactive astrocytes may represent main cortical or hippocampal cells in which Ten-2 or ADGRL1 immunoreactivity are upregulated after SE induction using the LiCl-pilocarpine model of epilepsy.

Our present study suggests that teneurin/TCAP/ADGRL coupling may be associated with neuroprotection during CNS damage and necrosis. Previous studies indicate that teneurins are expressed during neurogenesis where their functional roles are primarily related to neuronal migration, axonal orientation, cell differentiation, myelination and development and structural maintenance of synapses (Minet et al., 1999; Tucker and Chiquet-Ehrismann, 2006; Silva et al., 2011; Suzuki et al., 2012; Mosca, 2015; Antinucci et al., 2016; Tucker, 2018). However, numerous studies support a role of the teneurins during CNS damage. For instance, Ten-2 was re-expressed in external tufted cells during regeneration of olfactory sensory neurons, suggesting for the first time that this protein can be involved in CNS neuroplasticity (Otaki and Firestein, 1999). One study, using a rat model for ischemic stroke (middle cerebral artery occlusion), showed that the Ten-2 gene was one of the 50 most up-regulated genes in reactive astrocytes from mouse brain (Zamanian et al., 2012). Moreover, Ten-2 gene expression was up-regulated in human frontal cortex samples with amyotrophic lateral sclerosis (Andrés-Benito et al., 2017). Particularly, astrocytes differentiated from induced pluripotent stem cells (iPSCs) collected from patients with Alexander disease showed that Ten-2 was one of the most up-regulated genes compared to astrocytes differentiated from healthy patients (Kondo et al., 2016). Alexander disease is a catastrophic illness characterized, in part, by a significant rise of the mutated GFAP gene expression present in reactive astrocytes distributed throughout the CNS (Olabarria and Goldman, 2017; Sosunov et al., 2018). Furthermore, the Ten-4 gene was up-regulated in glutamate/H_2_O_2_-mediated oxidative stress-induced cell toxicity in Neuro-2a cells after treatment with *Anacardium occidentale* leaf extracts and, when knocked down by small interfering RNA (siTen-4), it reduced neurite outgrowth, suggesting the participation of Ten-4 in neurogenesis and neuroprotective actions (Duangjan et al., 2021). Finally, recent studies indicate that mutations or variants in the ADGRL1 gene are also associated with a range of CNS disorders, such as delayed speech development, intellectual disability, autism spectrum disorder, attention-deficit/hyperactivity disorder, as well as seizure disorders or epilepsy (Vitobello et al., 2022; Lei et al., 2025). ADGRL1 haploinsufficiency in humans and in heterozygous ADGRL1 null allele in mice were correlated with behavioral and neurological impairments, besides dysmorphologies or systemic pathologies and less frequently with epilepsy (Vitobello et al., 2022). Mutations in the GPCR-autoproteolysis inducing (GAIN) domain of the ADGRL1 protein were correlated with some cases of epileptic encephalopathy and genetic epilepsy with febrile seizures plus (GEFS+) (Lei et al., 2025). Taking together, these data support the suggestion that Ten/TCAP/ADGRL expression by reactive astrocytes may also be part of certain mechanisms activated after seizure disorders or epileptic events to protect the CNS.

Teneurins are complex proteins, and it is not clear which parts of the protein are associated with its actions in neurological disorders. Teneurins have several splice variants and points of cleavage located in the intra or extracellular domain (Lovejoy et al., 2006; Silva et al., 2011; Tucker et al., 2012; Vysokov et al., 2016; Vysokov et al., 2018). The cleavage at the extracellular end of the C-terminal domain results in small peptides of 40–41 amino acids named TCAP1-4, which exhibit bioactive properties (Qian et al., 2004; Wang et al., 2005; Lovejoy et al., 2006; Woelfle et al., 2015; Woelfle et al., 2016). Several studies indicate that TCAP-1 can exhibit neuroprotective and/or neuroplastic activities, as well as reduce stress-induced behaviors associated with anxiety, addiction and depression, reinstatement of cocaine-seeking and increase the glucose metabolism in neurons (Al Chawaf et al., 2007; Trubiani et al., 2007; Tan et al., 2009; Tan et al., 2011; Erb et al., 2014; Hogg et al., 2018). Particularly, TCAP-1 may play a neuroprotective role during periods of pH stress in immortalized hypothalamic neurons, suggesting that this neuropeptide protects neurons during chemical stress, such as hypoxia and ischemia (Trubiani et al., 2007). The present study also revealed the increase of TCAP-2 gene expression in the cerebral cortex and CA3 region of the hippocampus by real-time PCR. Moreover, a significant increase of TCAP-2 gene expression in the cerebral cortex after mechanical brain injury in adult rats has also been reported (Tessarin et al., 2019). Unfortunately, there is no available antibody targeting TCAP-2 specifically. However, it is plausible that Ten-2 and TCAP-2 are expressed simultaneously in the same reactive astrocytes after SE, as their gene expression oscillations were similar among the experimental groups, and previous data show that only TCAP-1 and TCAP-3 can be translated separately from Ten-1 and Ten-3 mRNA, respectively (Chand et al., 2013; Woelfle et al., 2015). However, it is possible that TCAP-2 can also be released from Ten-2, as there are amino acid motifs susceptible to proteolysis upstream the TCAP-2 sequence, as shown in studies focusing on the latrophilin-1-associated synaptic surface organizer (Lasso), a Ten-2 splice variant, and its additional bioactive peptides (Silva et al., 2011; Vysokov et al., 2016). Additional *in vivo* and *in vitro* studies are necessary to ascertain whether TCAP-2 itself exerts special biological activities, as previously demonstrated for TCAP-1.

Our present data also support the role of ADGRL with respect to neural injury. There is an interaction between Teneurin/TCAP and its endogenous receptor ADGRL where the latter may function as a cognate receptor to the former. ADGRL belongs to a family of postsynaptic Adhesion G Protein-Coupled Receptors (aGPCR) composed of ADGRL1, ADGRL2 and ADGRL3, also known as latrophilins (LPHNs) (Silva and Ushkaryov, 2010). ADGRL1 is expressed mainly in the brain, acting in neural circuit formation and neuronal migration, mediated by heterophilic interactions with teneurins, TCAP, neurexin and fibronectin and leucine-rich transmembrane proteins (FRLT) (Silva and Ushkaryov, 2010; Silva et al., 2011; Woelfle et al., 2015; Husić et al., 2019; Moreno-Salinas et al., 2019; Sando et al., 2019). Teneurin and ADGRL interaction was characterized not only by intercellular adhesion in the synapsis, but it can promote intracellular signaling in neurons, modulating calcium levels and/or cAMP pathways (Silva et al., 2011; Li et al., 2018; Ushkaryov et al., 2019). Lasso can interact mainly with ADGRL1, modulating the calcium influx in the presynaptic region (Silva et al., 2011). *In vitro* analysis of cerebellar immortalized astrocytes showed that this type of cell expresses TCAP-1-4 and this cell lineage responds to the treatment of synthetic TCAP-1, producing a significant increase in intracellular calcium, 3 and 6 min after TCAP-1 treatment (Tessarin et al., 2019). The TCAP sequence of teneurin is a potential domain for ADGRL interaction for cell stabilization and functional roles (Husić et al., 2019). Our current study showed a significant increase of ADGRL1-LI-reactive astrocytes, principally 2 and 5 days after SE. The colocalization of Ten-2 and ADGRL1 in the same reactive astrocytes shown in the present study could indicate self-stimulation or self-inhibition mechanisms in these cells through this signaling system, promoting a pivotal role in the maintenance of homeostasis of the brain, after deleterious effects of SE induction by the LiCl-pilocarpine model. Various studies indicated that astrocyte activity is also intimately involved in pro-convulsive or anti-convulsive effects in epilepsy disorders (Wetherington et al., 2008; Losi et al., 2012; Vargas-Sánchez et al., 2018; Sanz and Garcia-Gimeno, 2020; Müller et al., 2020; Verhoog et al., 2020; Heuser and Enger, 2021; Hayatdavoudi et al., 2022; Liu et al., 2022; Purnell et al., 2023). Astrocytes release gliotransmitters through tripartite synapses (distal cell extension of astrocytes close to synapse cleft), as glutamate, gamma-aminobutyric acid (GABA), ATP, D-serine and several other kinds of neuromodulators that can act in neurons modulating the synaptic functions in neurons (Araque et al., 1999; Araque et al., 2014; Gundersen et al., 2015; Harada et al., 2016; Mitterauer, 2015; Covelo and Araque, 2016). Under these conditions, the influence of astrocytes on the tripartite synapse may be induced in response to neuronal hyperactivity as what occurs in the epilepsy model induced by LiCl-pilocarpine, which results in toxicity mediated by calcium increase in the extracellular space (Scorza et al., 2009). Likewise, it is possible that the interaction between Ten-2/TCAP-2 and ADGRL1 in reactive astrocytes could be related to calcium influx. This calcium signaling can modulate the synapse functions in hyperactive neurons in several areas of the CNS, contributing to plasticity and neuronal repair, once reactive astrocytes release paracrine factors to protect the brain and help maintain neuronal networks (Sofroniew and Vinters, 2010; Sofroniew, 2014; Liu and Chopp, 2016; Linnerbauer and Rothhammer, 2020; Chiareli et al., 2021). It is also suitable to discuss the possibility of GABAergic astrocytes releasing this inhibitory neurotransmitter through calcium signaling in the tripartite synapse, to decrease the activity of excitatory neurons during acute and chronic phases of epilepsy or seizure disorder (Liu et al., 2022). In this protective mechanism to inhibit neuronal hyperexcitability, the activation of the Ten-2/TCAP-2/ADGRL1 signaling system could be modulating calcium signaling in reactive astrocytes, collaborating with GABA secretion from these cells.

Additional cell–cell interaction mediated between Ten-2/TCAP-2 and ADGRL1 is also possible since adjacent Ten-2-LI and ADGRL1-LI reactive astrocytes showed cell prolongments contacting or interdigitating with one another. For example, cell prolongments from one reactive astrocyte expressing Ten-2/TCAP-2 and ADGRL1 can interact with adjacent cell prolongments with ADGRL1 and Ten-2/TCAP-2 from other reactive astrocytes, like heterophilic or homophilic interactions. Astrocytes themselves communicate with each other by gap junctions facilitating the traffic of glucose, metabolites and several important types of molecules which circulate among nearby cells and/or distant cells as syncytial networks, collaborating with brain homeostasis, as well as energy metabolism (Sofroniew and Vinters, 2010; Sofroniew, 2014; Linnerbauer and Rothhammer, 2020; Chiareli et al., 2021). These functions are upregulated or downregulated in brain diseases (Harada et al., 2016; Covelo and Araque, 2016; Liu and Chopp, 2016; Kielian and Esen, 2004; Liddelow et al., 2017; Linnerbauer and Rothhammer, 2020). This way, the Ten-2/TCAP-2/ADGRL1 upregulation in reactive astrocytes may also contribute to morphological and functional coupling among these cells to restore local homeostasis, as well as to restrict and isolate lesioned areas.

The colocalization of Ten2 and ADGRL1 in the same reactive astrocytes in the current study could indicate a possible self-modulating mechanism, as previously mentioned. Bioactive peptides generated by Ten-2 proteolysis in reactive astrocytes can interact with ADGRL1, like an autocrine stimulation, activating certain cell parameters to support homeostasis of the cerebral cortex and hippocampus after the harmful effects evoked by persistent hyperexcitability of neurons induced by LiCl-pilocarpine. Pilocarpine induces neuronal stress and death where one of these mechanisms occurs through the activation of NMDA receptors by glutamate release in synapses, leading to calcium influx in the neurons, thus activating many enzymatic systems that result in neuronal apoptosis and necrosis (Scorza et al., 2009). During these processes, calcium intracellular homeostasis mechanisms are activated in the neurons to protect them from metabolic and functional stress by reducing intracellular calcium using Ca^2+^ATPases, such as sarcoendoplasmic reticulum (SR) calcium transport ATPase (SERCA) and plasma membrane Ca^2+^ ATPase (PMCA) (Scorza et al., 2009). PMCA activity allows calcium efflux from the neurons to the extracellular space, and this enzyme activity is evident after seizures induced by pilocarpine in acute and late phases after SE induction (Scorza et al., 2009). A previous study indicated that TCAP-1 can bind in ADGRL, decreasing cytosolic levels of Ca^2+^ in neurons, due to the efflux of this ion through calcium channels coupled in the cell membrane and/or due to the influx to mitochondria (Hogg et al., 2022). Hence, it is possible to hypothesize that Ten-2/TCAP-2/ADGRL1 upregulation in reactive astrocytes could be a protective mechanism to modulate calcium levels, minimizing neuronal death caused by calcium cytotoxicity induced by pilocarpine.

Ten-2 has also been reported to exert potential gene regulatory functions by acting as a transcription factor. A previous study demonstrated that homophilic interactions of Ten-2 trigger proteolytic cleavage of its intracellular domain (Bagutti et al., 2003). This cleaved intracellular fragment is subsequently translocated to the cell nucleus, where it colocalizes with promyelocytic leukemia (PML) protein within nuclear bodies and represses Zic-1–mediated transcription (Bagutti et al., 2003). Therefore, it cannot be excluded that Ten-2 may play a role in reactive astrocytes by modulating gene expression.

It has been reported that there is a significant impairment in the BBB permeability, a few minutes after induced SE, using either kainic acid or pilocarpine. Thus, facilitating the influx of proteins from blood to brain extracellular compartment and vice-versa. This condition may remain for several days in regions such as the hippocampus, entorhinal cortex, amygdala, and thalamus (Lassmann et al., 1984; Handforth and Treiman, 1995; Kim et al., 2010; Ndode-Ekane et al., 2010; Michalak et al., 2013; van Vliet et al., 2015). The endfeet processes of astrocytes are a fundamental part of the BBB, essentially involved in the maintenance and regulation of permeability at the interface of the circulatory system and nervous tissue in healthy conditions and in many types of CNS damage/injuries, including stroke, infection, and epilepsy (Janzer and Raff, 1987; Cavaglia et al., 2001; del Zoppo and Milner, 2006; Sofroniew and Vinters, 2010; Chodobski et al., 2011; Sofroniew, 2014; Serlin et al., 2015; Breuer et al., 2017; Giovannoni and Quintana, 2020; Reiss et al., 2023; Verkhratsky et al., 2023; Han et al., 2024). Previous studies have shown heterophilic interactions between teneurin and dystroglycan in the synthesis regulation of type IV collagen in the basal membrane of *Caenorhabditis elegans* (Trzebiatowska et al., 2008; Topf and Chiquet-Ehrismann, 2011), and the co-localization between Ten-1/TCAP-1 and α*β* dystroglycan in testes of adult rats, possibly regulating testicular size and testosterone synthesis (Chand et al., 2014). In addition, the colocalization of TCAP-1 and β-dystroglycan was demonstrated in hippocampal cells, promoting the organization of the cytoskeleton (Chand et al., 2012). Interestingly, β-dystroglycan is down regulated in the astrocytes endfeet noticed from *in vivo* cerebral cortex slices under zero magnesium (0 Mg2^+^), a brain slice model of epilepsy, which induces epileptiform discharges (Gondo et al., 2014). Ten-2-/ADGRL1-LI reactive astrocyte profiles projecting their cell extensions to blood vessels in the cerebral cortex and hippocampus was frequently seen in the current study. Thus, it can be hypothesized that reactive astrocytes can also be upregulating the Ten/TCAP/ADGRL1 complex system to minimize BBB permeability impairment, as a compensatory mechanism for possible dystroglycan down-regulation, during plasm extravasation in epilepsy/seizure disorders.

Finally, in several animal models of epilepsy, the presence of neuropathological changes with neuronal degeneration is present in cortical regions, such as the olfactory, frontal, temporal, parietal, entorhinal and perirhinal cortex, as well as in the hippocampal complex and in the thalamus (Turski et al., 1983; Clifford et al., 1987; Wang et al., 2008; Scholl et al., 2013). Previous studies indicate that teneurin and TCAP are involved with possible neuroprotection and neuroplasticity (Trubiani et al., 2007; Woelfle et al., 2016; Dodsworth and Lovejoy, 2022). Supporting this neuroprotective activity of the Ten/TCAP-/ADGRL1 system, the current study showed that the cerebral cortex, CA3 subregion of the hippocampus and several other CNS regions with neuronal degeneration, evidenced by Fluoro-Jade C staining technique, were encircled with Ten-2/ADGRL1 reactive astrocytes. Interestingly, the up-regulation of Ten-2, TCAP-2 and ADGRL1 were noticed only 48 h after SE with a peak at 5 days after SE, coinciding with the increase of anti-inflammatory markers involved in neuroprotection. This is different from the first hours after SE induction (acute phase), when a plethora of proinflammatory and proconvulsive substances, such as IL-1β, TNF and IL-6 are released mainly from microglia (Shapiro et al., 2008; Liddelow et al., 2017; Sano et al., 2021). Reactive astrocytes are classified into two types, based on their activation mechanisms and the cytokines they secrete: type A2, which shows neuroprotective functions, and type A1, which displays neurotoxic functions (Sofroniew, 2014; Liddelow and Barres, 2017; Fan and Huo, 2021). Further studies are important to confirm if Ten-2-/ADGRL1-LI reactive astrocytes belong to type A2, which display neuroprotective phenotypes, different from A1 which secrete various proinflammatory substances or if they belong to both subtypes.

### 4.3 Limitations of study and future directions

Although the present study demonstrated that the Ten-2/TCAP-2/ADGRL1 system is upregulated in reactive astrocytes in various CNS regions following SE induced by the lithium-pilocarpine model in rats, several questions remain to be explored, limiting the scope of our current findings. For instance, single-cell quantitative RT-PCR would allow a more precise assessment of the proportion of Ten-2, TCAP-2, and ADGRL1 gene expression in individual neural cell types after SE induction, like a recent study by Wang and colleagues (Wang et al., 2025).

The data in the present study are limited to male adult rats, and therefore our results cannot be directly extended to female adult rats. A previous study compared male and female adult rats using the LiCl–pilocarpine model to induce SE and follow-up for 90 days (Matovu and Cavalheiro, 2022). This study revealed significant sex-related differences in the frequency of spontaneous recurrent seizures, as well as in the topographical distribution of neuronal and non-neuronal cell death (Matovu and Cavalheiro, 2022). It is also known that some types of epilepsy are influenced by fluctuations in sex hormones, such as in catamenial epilepsy in drug-resistant epilepsy patients (Herzog, 2015; Reddy, 2016). In these cases, seizure frequency increases during the perimenstrual and peri-ovulatory periods (Herzog, 2015; Reddy, 2016). Furthermore, it has been suggested that seizure frequency increases during the perimenopausal period and decreases after menopause in women with epilepsy from earlier ages (Harden et al., 1999; Velíšková and Desantis, 2013).

Ten-2 displays complex biochemical and functional properties. It primarily acts as a transmembrane protein but can undergo proteolytic cleavage, releasing bioactive peptides into the extracellular space that may influence neighboring neuronal cells (Silva et al., 2011; Tucker and Chiquet-Ehrismann, 2006; Mosca, 2015; Tucker et al., 2012; Tucker, 2018). Ten-2 splice variants have also been noticed (Silva et al., 2011; Tucker et al., 2012). Its intracellular domain can be cleaved and translocated to the nucleus, where it may function as a transcriptional regulator (Bagutti et al., 2003). These diverse modes of action make it challenging to determine whether the upregulation of the Ten-2/TCAP-2/ADGRL1 system observed in reactive astrocytes in the present study reflects its role as a membrane-associated protein mediating homophilic or heterophilic interactions, or as a paracrine signaling molecule, or as a transcriptional regulatory factor modulating astrocyte reactivity. Thus, studies using additional and/or novel methodologies are necessary to elucidate this concern.

Determining whether the Ten-2/TCAP-2/ADGRL1 system is associated with type 2 reactive astrocytes, which are known to exhibit neuroprotective properties, could provide important insights into the functional significance of this protein system in the epilepsy model used here (Sofroniew, 2014; Liddelow and Barres, 2017; Fan and Huo, 2021). Additional experimental models of SE and chronic epilepsy may also clarify or support whether the upregulation of the Ten-2/TCAP-2/ADGRL1 system in reactive astrocytes represents a general response to neuronal injury caused by sustained neuronal hyperexcitability (Reddy and Kuruba, 2013; Kandratavicius et al., 2014; Wang et al., 2022).

Finally, the LiCl–pilocarpine model of epilepsy can be particularly useful for evaluating synaptic plasticity and circuitry reorganization in the hippocampal complex during both short-term (ictal and post-ictal phases) and long-term periods (latency and spontaneous recurrent seizures periods). All teneurins and ADGRL paralogues are known to be expressed in the hippocampal complex, mainly in neurons (Liakath-Ali et al., 2024). Moreover, it is well established that these proteins play essential roles in the development and maintenance of synapses through homophilic or heterophilic interactions between pre- and postsynaptic compartments in both inhibitory and excitatory synapses (Silva et al., 2011; Mosca, 2015; Tucker, 2018; Sando et al., 2019; Cheung et al., 2022; Südhof, 2025; Zhang et al., 2025). Thus, future studies focusing on teneurin-ADGRL interactions using the LiCl–pilocarpine model may be suitable to reveal key insights into how this protein system contributes to synapse formation and neuroplasticity.

## 5 Conclusion

The present study showed that Ten-2/TCAP-2 and its potential receptor, ADGRL1, were up-regulated in reactive astrocytes in the cerebral cortex and hippocampus of adult male rats, mostly two and five days after *status epilepticus,* using the LiCl-pilocarpine model of epilepsy. Further studies using novel methodological approaches are necessary to investigate the detailed functions of the Ten-2/TCAP-2/ADGRL1 system and whether this signaling system can be a potential therapeutic target to prevent or minimize the harmful effects in brain disorders.

## Data Availability

The datasets presented in this study can be found in online repositories. The names of the repository/repositories and accession number(s) can be found https://repositorio.unesp.br/entities/publication/45dcc187-6cca-4c10-8670-ebda2c0bc91c, 11449/191385. https://repositorio.unesp.br/server/api/core/bitstreams/9018051c-6fe3-4382-9cf4-4c4b2ffd4912/content.

## Supplementary material

SUPPLEMENTARY FIGURE S1 Nissl **(A)** and indirect immunoperoxidase **(B)** stains showing some thalamic nuclei encircled by Ten-2-Li reactive astrocytes in animal from EG5. 3V, third ventricle; AHC, hypothalamic anterior area; AM, anteromedial thalamic nucleus; Arc, arcuate nucleus; D, dorsal; DG, dentate gyrus; f, fornix; L, lateral; LH, lateral hypothalamic area; LHb, lateral habenular nucleus; M, medial; MHb, medial habenular nucleus; MDM, mediodorsal thalamic nucleus, medial; mt, mammillothalamic tract; PaMP, paraventricular nucleus, medial parvicellular; PVA, paraventricular thalamic nucleus; Re, reuniens thalamic nucleus; opt, optic tract; SO, supraoptic nucleus; Sub, submedius thalamic nucleus; V, ventral.

SUPPLEMENTARY FIGURE S2 Nissl **(A)** and indirect immunoperoxidase **(B–D)** stains showing Ten-2-Li reactive astrocytes in animal from EG5. The inset boxes from panel **(B–D)** are highlighted in panel **(D)**. Observe in B-D intense presence of Ten-2-LI reactive astrocytes (blue arrows) encircling strong neuronal degeneration in the piriform and entorhinal cortices. In panel **(B–D)** are also shown Ten-2-LI reactive astrocytes in the central amygdaloid nucleus (white arrow). Yellow asterisk is showing strong neuronal degeneration area. 3V, third ventricle; AA, anterior amygdaloid area; ACo, anterior cortical amygdaloid nucleus; AH, hypothalamic anterior area; Arh, arcuate nucleus; BMA, basomedial amygdaloid nucleus; CA1, cornu Ammonis 1 of the hippocampus; CA3, cornu Ammonis 3 of the hippocampus; CeA, central amygdaloid nucleus; CPu, caudate putamen; DG, dentate gyrus; f, fornix; fi, fimbria of hippocampus; ic, internal capsule; LGP, lateral globus pallidus; LH, lateral hypothalamic area; M, motor cortex; mt, mammillothalamic tract; opt, optic tract; ox, optic chiasm; Pa, paraventricular hypothalamic nucleus; Pir, piriform cortex; PVA, paraventricular thalamic nucleus; Re, reuniens thalamic nucleus; RSG, retrosprenial granular cortex; S1, primary somatosensory cortex; S2, secondary somatosensory cortex; Sm, stria medullaris of thalamus; SO, supraoptic nucleus; St, stria terminalis; Rf, rhinal fissure; LV, lateral ventricle.

SUPPLEMENTARY FIGURE S3 Double labeling of Ten-2-LI/Fluoro jade-C in CA1 **(A–A”)**, dentate gyrus **(B–B”)**, piriform cortex **(C–C”)**, amygdaloid complex **(D–D”)** and anterior thalamic nucleus **(E–E”)** in a histologic section from an animal of EG5 group. Note that Ten-2-LI reactive astrocytes coincide with neurons stained with Fluoro-Jade C staining. 3V, third ventricle; AMG, amygdala; CA1, cornu Ammonis 1 of the hippocampus; DG, dentate gyrus; Pir, piriform cortex; Ten-2, teneurin-2.
