# Supplementary figures and images for: Teneurin-2 and related proteins in reactive astrocytes after status epilepticus induction in adult rats
Source: Front Neurosci. 2026 Mar 18;19:1670634. doi: 10.3389/fnins.2025.1670634 (PMC13038874; doi:10.3389/fnins.2025.1670634)

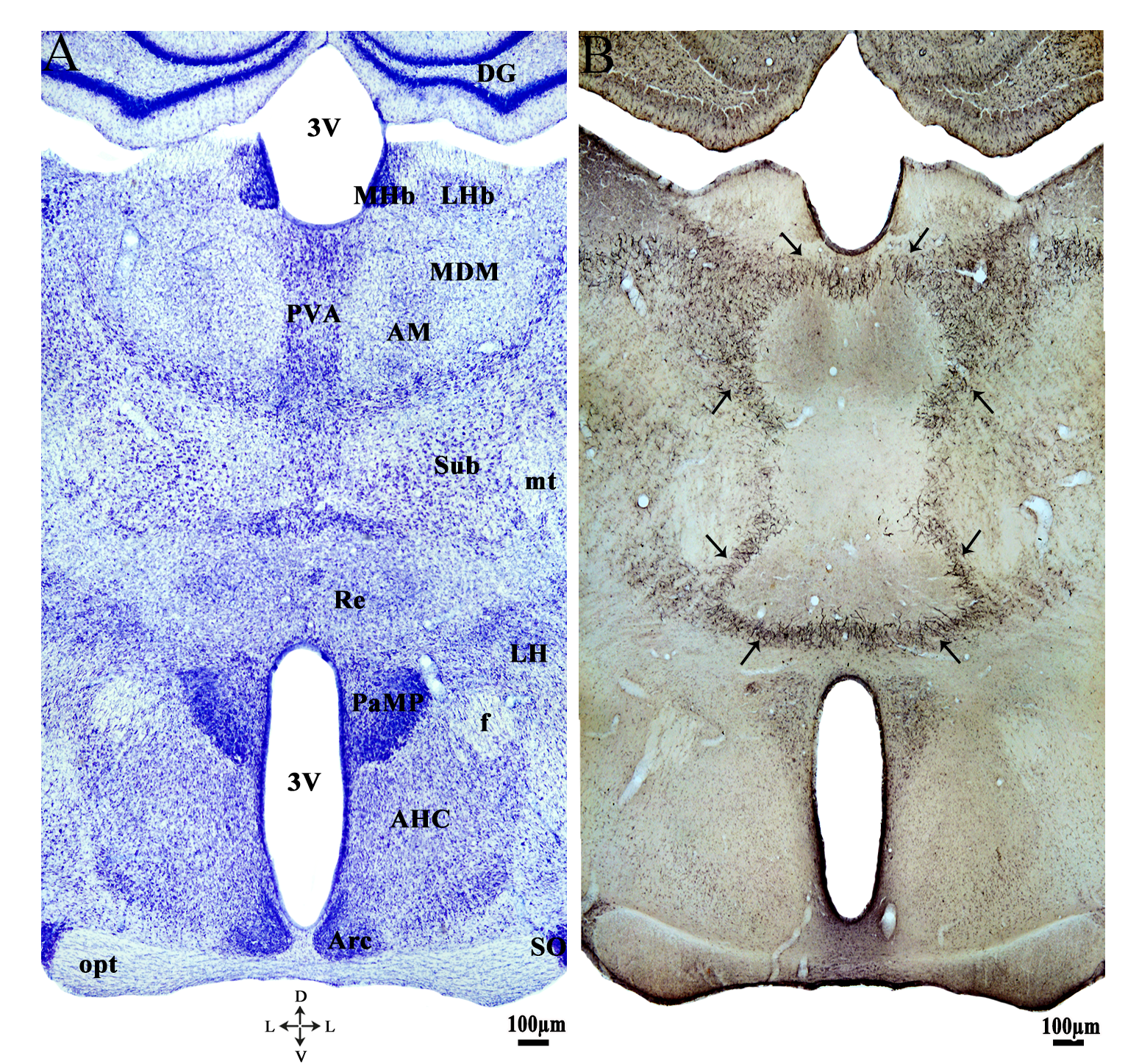

Supplement: SUPPLEMENTARY FIGURE S1 — Nissl (A) and indirect immunoperoxidase (B) stains showing some thalamic nuclei encircled by Ten-2-Li reactive astrocytes in animal from EG5. 3V, third ventricle; AHC, hypothalamic anterior area; AM, anteromedial thalamic nucleus; Arc, arcuate nucleus; D, dorsal; DG, dentate gyrus; f, fornix; L, lateral; LH, lateral hypothalamic area; LHb, lateral habenular nucleus; M, medial; MHb, medial habenular nucleus; MDM, mediodorsal thalamic nucleus, medial; mt, mammillothalamic tract; PaMP, paraventricular nucleus, medial parvicellular; PVA, paraventricular thalamic nucleus; Re, reuniens thalamic nucleus; opt, optic tract; SO, supraoptic nucleus; Sub, submedius thalamic nucleus; V, ventral. [file Image_1.TIF]

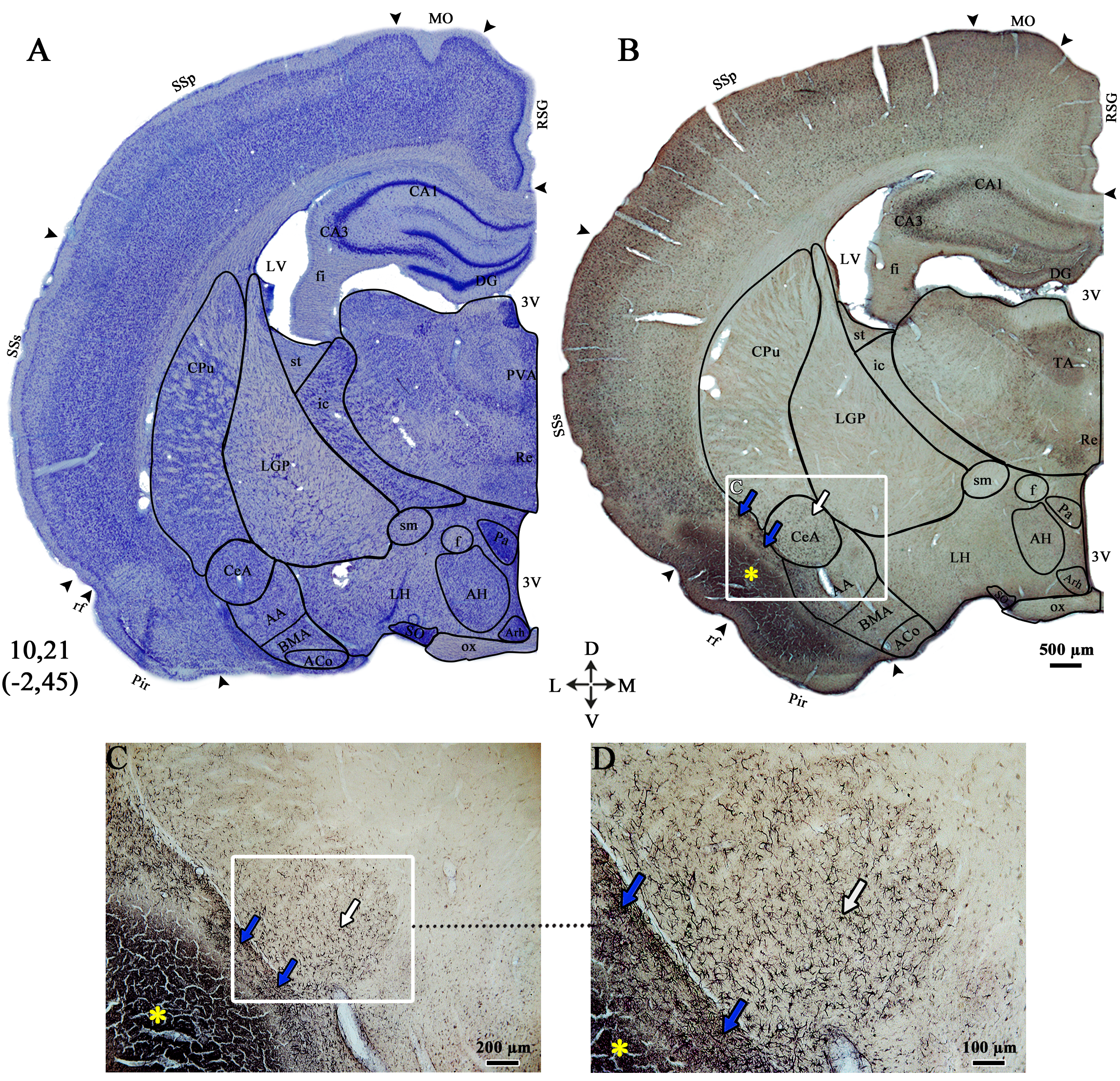

Supplement: SUPPLEMENTARY FIGURE S2 — Nissl (A) and indirect immunoperoxidase (B–D) stains showing Ten-2-Li reactive astrocytes in animal from EG5. The inset boxes from panel (B–D) are highlighted in panel (D). Observe in B-D intense presence of Ten-2-LI reactive astrocytes (blue arrows) encircling strong neuronal degeneration in the piriform and entorhinal cortices. In panel (B–D) are also shown Ten-2-LI reactive astrocytes in the central amygdaloid nucleus (white arrow). Yellow asterisk is showing strong neuronal degeneration area. 3V, third ventricle; AA, anterior amygdaloid area; ACo, anterior cortical amygdaloid nucleus; AH, hypothalamic anterior area; Arh, arcuate nucleus; BMA, basomedial amygdaloid nucleus; CA1, cornu Ammonis 1 of the hippocampus; CA3, cornu Ammonis 3 of the hippocampus; CeA, central amygdaloid nucleus; CPu, caudate putamen; DG, dentate gyrus; f, fornix; fi, fimbria of hippocampus; ic, internal capsule; LGP, lateral globus pallidus; LH, lateral hypothalamic area; M, motor cortex; mt, mammillothalamic tract; opt, optic tract; ox, optic chiasm; Pa, paraventricular hypothalamic nucleus; Pir, piriform cortex; PVA, paraventricular thalamic nucleus; Re, reuniens thalamic nucleus; RSG, retrosprenial granular cortex; S1, primary somatosensory cortex; S2, secondary somatosensory cortex; Sm, stria medullaris of thalamus; SO, supraoptic nucleus; St, stria terminalis; Rf, rhinal fissure; LV, lateral ventricle. [file Image_2.TIF]

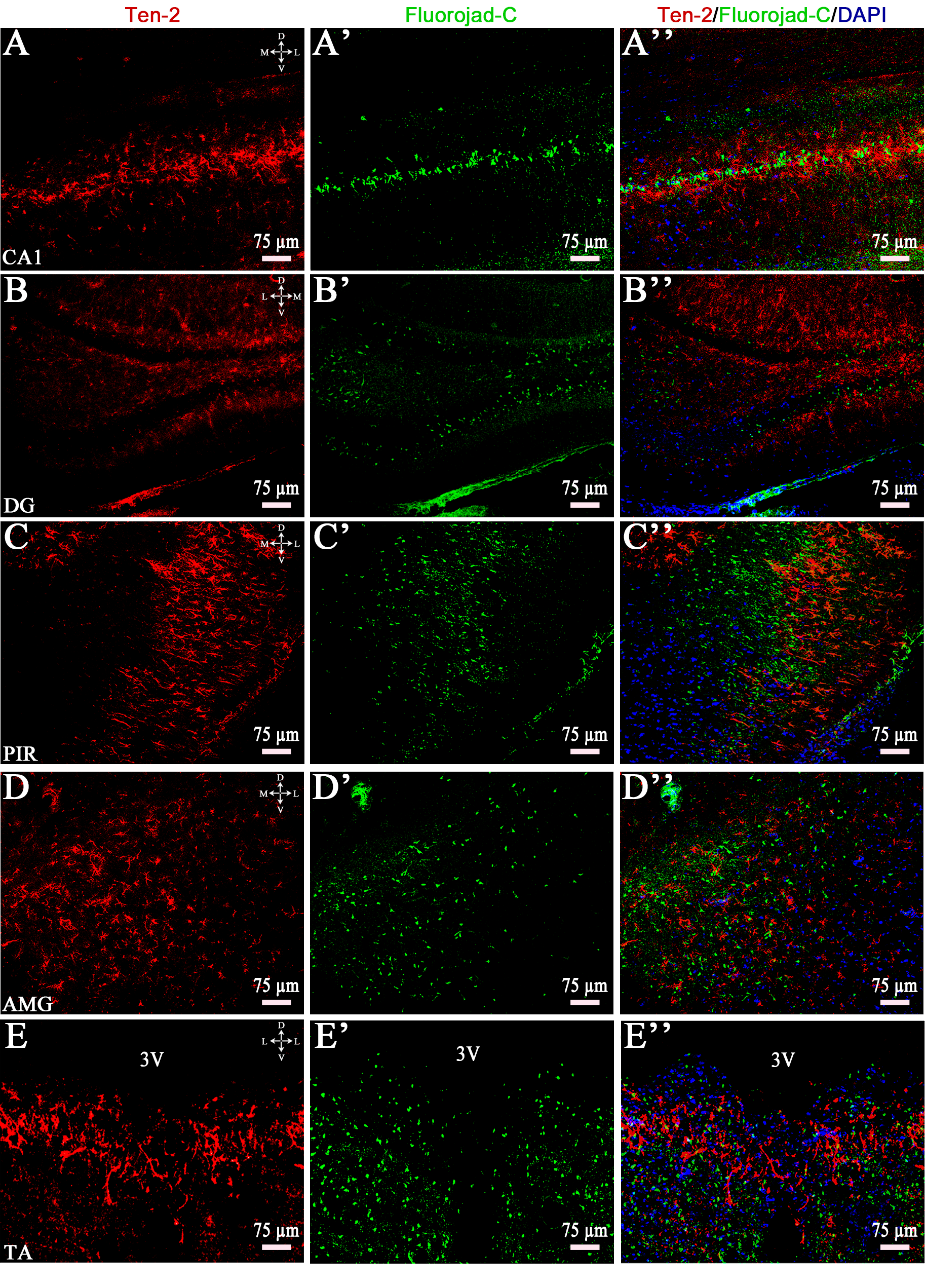

Supplement: SUPPLEMENTARY FIGURE S3 — Double labeling of Ten-2-LI/Fluoro jade-C in CA1 (A–A”), dentate gyrus (B–B”), piriform cortex (C–C”), amygdaloid complex (D–D”) and anterior thalamic nucleus (E–E”) in a histologic section from an animal of EG5 group. Note that Ten-2-LI reactive astrocytes coincide with neurons stained with Fluoro-Jade C staining. 3V, third ventricle; AMG, amygdala; CA1, cornu Ammonis 1 of the hippocampus; DG, dentate gyrus; Pir, piriform cortex; Ten-2, teneurin-2. [file Image_3.TIF]
